# Reversal of viral and epigenetic HLA class I repression in Merkel cell carcinoma

**DOI:** 10.1172/JCI151666

**Published:** 2022-07-01

**Authors:** Patrick C. Lee, Susan Klaeger, Phuong M. Le, Keegan Korthauer, Jingwei Cheng, Varsha Ananthapadmanabhan, Thomas C. Frost, Jonathan D. Stevens, Alan Y.L. Wong, J. Bryan Iorgulescu, Anna Y. Tarren, Vipheaviny A. Chea, Isabel P. Carulli, Camilla K. Lemvigh, Christina B. Pedersen, Ashley K. Gartin, Siranush Sarkizova, Kyle T. Wright, Letitia W. Li, Jason Nomburg, Shuqiang Li, Teddy Huang, Xiaoxi Liu, Lucas Pomerance, Laura M. Doherty, Annie M. Apffel, Luke J. Wallace, Suzanna Rachimi, Kristen D. Felt, Jacquelyn O. Wolff, Elizabeth Witten, Wandi Zhang, Donna Neuberg, William J. Lane, Guanglan Zhang, Lars R. Olsen, Manisha Thakuria, Scott J. Rodig, Karl R. Clauser, Gabriel J. Starrett, John G. Doench, Sara J. Buhrlage, Steven A. Carr, James A. DeCaprio, Catherine J. Wu, Derin B. Keskin

**Affiliations:** 1 Department of Medical Oncology, Dana-Farber Cancer Institute, Boston, Massachusetts, USA.; 2 Harvard Medical School, Boston, Massachusetts, USA.; 3 Broad Institute of MIT and Harvard, Cambridge, Massachusetts, USA.; 4 Department of Statistics, University of British Columbia, Vancouver, British Columbia, Canada.; 5 BC Children’s Hospital Research Institute, Vancouver, British Columbia, Canada.; 6 Department of Medicine, Brigham and Women’s Hospital, Boston, Massachusetts, USA.; 7 Department of Molecular, Cellular and Biomedical Sciences, University of New Hampshire, Durham, New Hampshire, USA.; 8 Program in Virology, Graduate School of Arts and Sciences, Harvard University, Cambridge, Massachusetts, USA.; 9 Department of Pathology, Brigham and Women’s Hospital, Boston, Massachusetts, USA.; 10 Translational Immunogenomics Laboratory, Dana-Farber Cancer Institute, Boston, Massachusetts, USA.; 11 Section for Bioinformatics, Department of Health Technology, Technical University of Denmark, Lyngby, Denmark.; 12 Center for Genomic Medicine, Copenhagen University Hospital, Rigshospitalet, Copenhagen, Denmark.; 13 Department of Biomedical Informatics, Harvard Medical School, Boston, Massachusetts, USA.; 14 Department of Cancer Biology and the Linde Program in Cancer Chemical Biology, Dana-Farber Cancer Institute, Boston, Massachusetts, USA.; 15 Department of Biological Chemistry and Molecular Pharmacology,; 16 Department of Immunology, and; 17 Department of Systems Biology and Laboratory of Systems Pharmacology, Harvard Medical School, Boston, Massachusetts, USA.; 18 Center for Immuno-Oncology and; 19 Department of Data Science, Dana-Farber Cancer Institute, Boston, Massachusetts, USA.; 20 Department of Computer Science, Metropolitan College, Boston University, Boston, Massachusetts, USA.; 21 Department of Dermatology, Brigham and Women’s Hospital, Harvard Medical School, Boston, Massachusetts, USA.; 22 Merkel Cell Carcinoma Center of Excellence, Dana-Farber/Brigham Cancer Center, Boston, Massachusetts, USA.; 23 Laboratory of Cellular Oncology, Center for Cancer Research, National Cancer Institute, NIH, Bethesda, Maryland, USA.

**Keywords:** Oncology, MHC class 1, Proteomics, Skin cancer

## Abstract

Cancers avoid immune surveillance through an array of mechanisms, including perturbation of HLA class I antigen presentation. Merkel cell carcinoma (MCC) is an aggressive, HLA-I–low, neuroendocrine carcinoma of the skin often caused by the Merkel cell polyomavirus (MCPyV). Through the characterization of 11 newly generated MCC patient-derived cell lines, we identified transcriptional suppression of several class I antigen presentation genes. To systematically identify regulators of HLA-I loss in MCC, we performed parallel, genome-scale, gain- and loss-of-function screens in a patient-derived MCPyV-positive cell line and identified MYCL and the non-canonical Polycomb repressive complex 1.1 (PRC1.1) as HLA-I repressors. We observed physical interaction of MYCL with the MCPyV small T viral antigen, supporting a mechanism of virally mediated HLA-I suppression. We further identify the PRC1.1 component USP7 as a pharmacologic target to restore HLA-I expression in MCC.

## Introduction

The therapeutic landscape of cancer treatment has been transformed by potent immunotherapeutic agents such as checkpoint blockade inhibitors. Despite their promise, the majority of cancer patients demonstrate an inadequate response. A more precise understanding of immune evasion is paramount to advancing immunotherapy, and one important mechanism of resistance is loss of human leukocyte antigen class I (HLA-I). The frequency of HLA-I loss can reach 80% in many cancers (1); it occurs through genomic or transcriptional alterations to class I antigen presentation machinery (APM) genes (2–4). HLA-I loss correlates with a worse prognosis and is a common mechanism of resistance to immunotherapy (4–8). The restoration of HLA-I expression, specifically in the case of transcriptional loss, represents an unmet therapeutic need and may synergize with existing immunotherapies. While interferon-γ (IFN-γ) is a known inducer of HLA-I, endogenous intratumoral IFN-γ is primarily produced by tumor-infiltrating lymphocytes (9, 10) and thus is closely linked to tumor HLA-I expression. Moreover, exogenous IFN-γ produces systemic side effects and may exert pro-tumorigenic effects as well (11). The development of targeted HLA-I–upregulating agents necessitates a better understanding of how cancers transcriptionally suppress class I APM genes. One intriguing model system to study this is Merkel cell carcinoma (MCC).

MCC is a rare but highly aggressive neuroendocrine carcinoma of the skin, caused by the Merkel cell polyomavirus (MCPyV) in approximately 80% of cases (12, 13). MCPyV^+^ MCC is a low–tumor mutational burden (low-TMB) subtype driven by 2 viral antigens: large T antigen (LT), which inactivates RB1 (14), and small T antigen (ST), which has numerous functions, including recruitment of MYCL, a MYC paralog, to chromatin-modifying complexes (15). By contrast, MCPyV^–^ MCC exhibits high TMB secondary to ultraviolet (UV) damage and almost invariably contains mutations in *TP53* and *RB1*. Notably, both subtypes of MCC exhibit low HLA-I expression, which has previously been observed by immunohistochemistry (IHC) in 84% of MCC tumors and confirmed in MCC cell lines (16, 17). However, HLA-I expression in MCC also appears to be highly plastic, as it can be upregulated in vitro by IFNs or histone deacetylase inhibitors (16, 17).

Existing MCC lines are limited in number, and several are poor representatives of primary tumors (18). We established an approach to consistently generate MCC patient-derived cell lines directly from tumor biopsies and patient-derived xenografts. We hypothesized that viral antigen–mediated signaling suppresses HLA-I surface expression in MCPyV^+^ MCC through regulatory pathways that may also be perturbed in MCPyV^–^ MCC and other cancers. We systematically characterized class I APM genes in 11 newly generated MCC lines through genomic and proteomic analysis. We then interrogated MCC lines through genome-scale gain- and loss-of-function screens for the restoration of HLA-I. These screens identified MYCL and the non-canonical Polycomb repressive complex 1.1 (PRC1.1) as regulators of HLA-I. We further demonstrate that pharmacologic inhibition of the PRC1.1 component USP7 can restore HLA-I expression.

## Results

### Reliable generation of MCC cell lines from primary patient samples.

**Figure 1:**
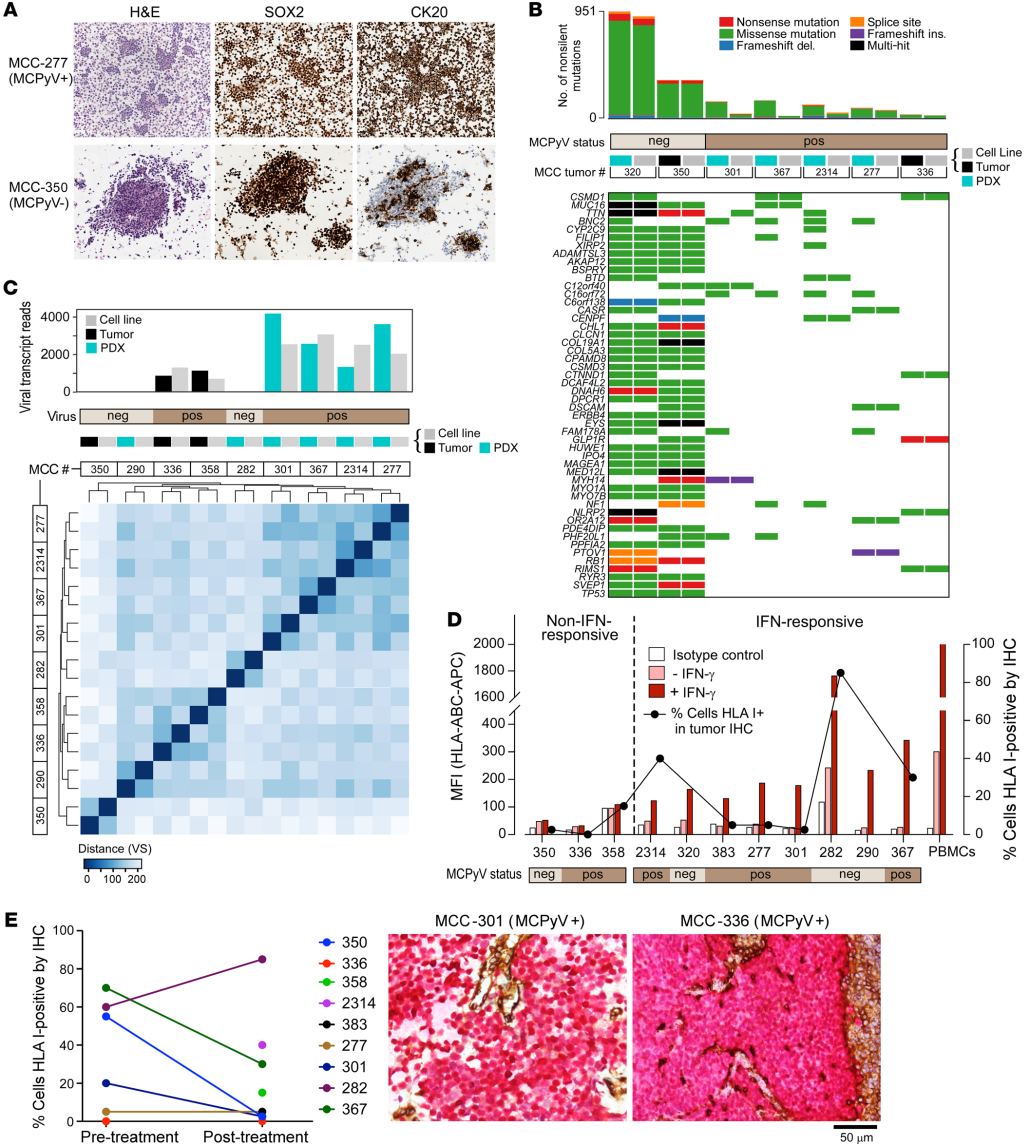
Generation of patient-derived MCC lines that exhibit classic MCC features and recapitulate the low HLA-I expression of their corresponding tumors. (**A**) IHC of 2 MCC lines stained for MCC markers SOX2 and CK20 (original magnification, ×20). (**B**) CoMut plot displaying the top 50 most frequently mutated genes across 7 MCC tumor and cell line pairs. (**C**) Unsupervised hierarchical clustering of RNA-Seq data from 9 MCC patient tumors and corresponding cell lines. Heatmaps were constructed using a distance matrix on variance-stabilizing (VS) transformed expression values. Top track: Quantification of transcript reads mapping to the MCPyV genome. (**D**) HLA-I flow cytometry in 11 MCC lines, both at baseline (pink bars) and in response to IFN-γ (red bars), compared with isotype control (white bars). The black line plot indicates the percentage of tumor cells positive for HLA-I by IHC of the original tumor. (**E**) IHC of MCC tumor archival samples. Left: Summary of the percentage of MCC cells that are HLA-I positive within available pretreatment (*n =* 6) and post-treatment (*n =* 9) tumor samples (see Table 1 for prior treatments). MCC cell lines were derived from post-treatment samples. Right: Representative IHC images of 2 HLA-I–low tumors, MCC-301 and MCC-336, stained for HLA class I (brown) with SOX2 costain (red) to identify MCC cells. Lymphocytes and endothelial cells served as internal controls that are SOX2 negative and HLA-I positive. Scale bar: 50 μm.

Since many established MCC lines have been multiply passaged in vitro and lack primary tumor material (19–22), we established an approach to generate our own MCC lines. Although MCC is typically cultured in RPMI 1640 medium, we hypothesized that a neuronal stem cell medium that we previously used to establish glioblastoma cell lines (23) would facilitate cell line establishment, based on MCC’s neuroendocrine histology and prior reports of successful MCC line generation with a neural crest stem cell medium (24). Of 5 medium formulations tested, NeuroCult NS-A Proliferation medium with growth factor supplementation consistently provided the highest in vitro growth rate, tripling cell numbers after 7 days in culture (Supplemental Figure 1A; supplemental material available online with this article; https://doi.org/10.1172/JCI151666DS1) and facilitating reliable growth of multiple MCC lines (Supplemental Figure 1B). Using this method, we established 11 cell lines directly from tumor biopsies (*n =* 4) or patient-derived xenografts (PDXs) (*n =* 7) (Table 1). Consistent with established MCC lines (25), these lines grew mostly in tight suspension clusters and stained positive for the MCC markers SOX2 and CK20, except for CK20 negativity in MCC-320 (Figure 1A and Supplemental Figure 1C). We determined that 7 of the 11 lines (63.6%) were MCPyV^+^ using ViroPanel (26) (Supplemental Figure 1D; and see Supplemental Methods).

We performed whole-exome sequencing on tumor DNA from 7 of 11 patients for whom matched cell line and germline DNA was available (Supplemental Table 1). MCPyV^–^ (*n =* 2) and MCPyV^+^ (*n =* 5) samples exhibited contrasting high (median 647 non-silent coding mutations per cell line, range 354–940) and low TMBs (median 40, range 18–73) (Figure 1B and Supplemental Table 1), respectively, as expected. The 2 analyzed MCPyV^–^ lines contained *RB1* and *TP53* mutations (Supplemental Table 1), consistent with previous studies (27, 28). A median of 94.4% of cell line mutations were detected in the corresponding primary samples (range 51%–100%), and tumor–cell line pairs associated closely based on their mutational profiles (Supplemental Figure 1E). Several PDX-derived tumor samples (Table 1) did exhibit higher mutational burdens than their corresponding cell lines (Figure 1B), likely due to murine cell contamination. Corresponding RNA sequencing (RNA-Seq) of available matched tumors and cell line pairs (Supplemental Table 1) detected MCPyV ST and LT antigen transcripts in all MCPyV^+^ samples (Figure 1C and Supplemental Figure 1D). Cell line transcriptomes associated most closely with corresponding parent tumors (mean pairwise Spearman’s correlation 0.92) (Figure 1C and Supplemental Figure 1F), rather than clustering by sample type, confirming that these lines faithfully recapitulate parent tumors.

We observed that 10 of 11 MCC lines strikingly exhibited low surface HLA-I by flow cytometry (Figure 1D), similarly to the well-studied MCPyV^+^ lines MKL-1 and WaGa (Supplemental Figure 2A). Three lines (MCC-336, -350, and -358) did not appreciably upregulate HLA-I after IFN-γ exposure (≤1.15-fold increase in MFI), whereas 8 lines exhibited at least a 2.5-fold increase (median 5.7, range 2.5–12.4). We further confirmed in 2 lines that IFN-α2b and IFN-β upregulated HLA-I (Supplemental Figure 2B), while IFN-γ also upregulated HLA-DR expression in the MCC-301 cell line (Supplemental Figure 2C).

These patient-derived cell line results were consistent with immunohistochemical (IHC) characterization of HLA-I on 9 parental tumors, in which the majority (6 of 9) displayed HLA-I–positive staining in less than 15% of tumor cells (Figure 1D and Supplemental Figure 2, D and E), as well as minimal HLA class II (Supplemental Figure 2F). The tumor-infiltrating CD8^+^ T cell density (median 56.6 cells/mm^2^, range 0–1031.8) was on par with previous reports for MCC (ref. 29 and Supplemental Figure 2G). Moreover, the availability of pre- and post-treatment formalin-fixed, paraffin-embedded tumor samples allowed us to assess temporal changes in HLA-I expression. The most common treatment was radiation with or without cisplatin and etoposide (Table 1). In 5 of 6 cases with available paired samples, post-treatment specimens demonstrated fewer HLA-I–positive cells than pretreatment specimens (Figure 1E), further implicating HLA-I loss as a mechanism of therapeutic resistance in MCC.

### MCC lines exhibit transcriptional downregulation of multiple class I genes and NLRC5 alterations.

**Figure 2:**
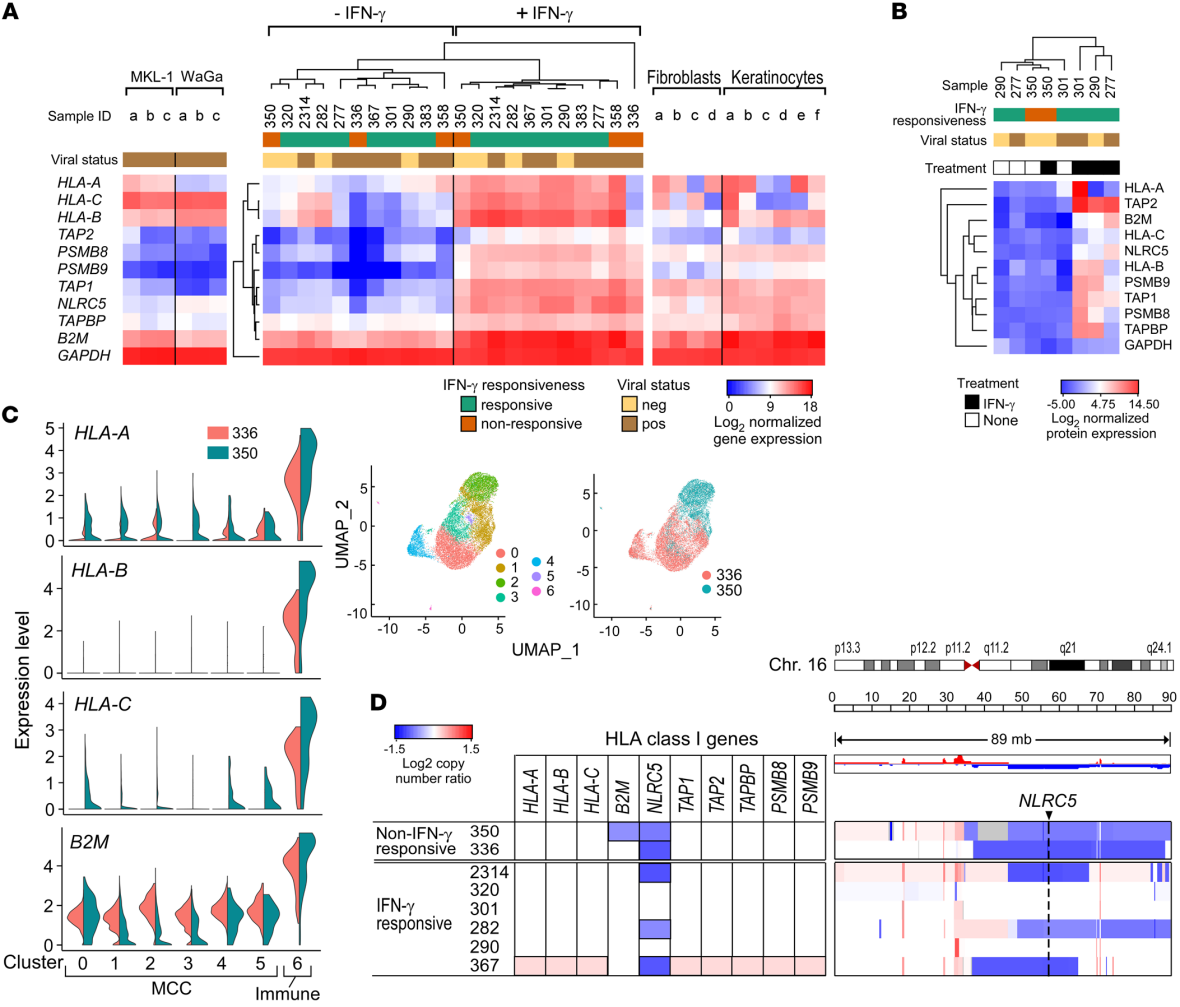
Transcriptional repression of multiple class I pathway genes and NLRC5 alterations underlie the loss of HLA-I surface expression in the panel of MCC lines. (**A**) RNA-Seq heatmaps of class I antigen presentation gene expression. Middle heatmap: Unsupervised clustering by Euclidean distance of the MCC cell line panel, with and without IFN-γ treatment. Left: Reference heatmap of MCC lines MKL-1 and WaGa. Right: Reference heatmap of epidermal keratinocytes and dermal fibroblasts. (**B**) Unsupervised clustering by Euclidian distance of protein expression values for class I genes, with and without IFN-γ treatment. (**C**) scRNA-Seq data from MCC-336 (MCPyV^+^) and MCC-350 (MCPyV^–^) fresh tumor samples. Right panel: UMAP (uniform manifold approximation and projection) visualization of all cells is displayed, colored by cluster (left) and by sample (right). Left panel: Expression levels of *HLA-A*, *-B*, and *-C* and *B2M* across all clusters (clusters 0–5, MCC cells; cluster 6, immune cells). (**D**) log_2_ copy number ratios for class I genes (left) and for chromosome 16 (right), where *NLRC5* is located.

To elucidate the mechanisms of HLA-I loss in these MCC cell lines, we performed an in-depth genomic and transcriptional characterization of all MCPyV^+^ and MCPyV^–^ lines for which material was available (Supplemental Table 1). To define class I APM transcriptional alterations, we evaluated the transcriptomes of all 11 MCC lines before and after IFN-γ stimulation. At baseline, the MCC lines exhibited low expression of *HLA-B*, *TAP1*, *TAP2*, *PSMB8*, and *PSMB9*, compared with control epidermal keratinocytes and dermal fibroblasts (30, 31), which are candidates for the cell of origin of MCPyV^–^ and MCPyV^+^ MCC (ref. 32 and Figure 2A). IFN-γ treatment markedly upregulated class I gene transcripts (Supplemental Figure 3A and Supplemental Table 1), a trend that was confirmed in matched proteomes in 4 MCC lines (Figure 2B). Non–IFN-γ–responsive lines (Figure 1D) exhibited variable defects, such as a relative decrease in IFN-induced *HLA-A*, *-B*, and *-C* mRNA upregulation (MCC-336) or *TAP2* and *PSMB8/9* upregulation (MCC-350) (Figure 2A), and global lack of IFN-induced HLA-I and IFN pathway upregulation at the protein level (MCC-350), including lack of STAT1 phosphorylation (Figure 2B and Supplemental Figure 3, B and C).

To investigate the heterogeneity in the HLA-I downregulation observed in our bulk RNA-Seq data, we performed droplet-based single-cell RNA-Seq on 2 fresh MCC biopsies (MCC-350 [MCPyV^–^] and MCC-336 [MCPyV^+^]). Within 15,808 cells (mean 4231.9 genes/cells) across both samples, 7 transcriptionally defined clusters were detected. *CD45*^+^ immune cells constituted cluster 6, while clusters 0–5 were MCC cells, identified by the expression of *SOX2*, *SYP*, and *ATOH1* (Figure 2C and Supplemental Figure 3D). All MCC clusters displayed nearly absent *HLA-B*, *TAP1/2*, *PSMB8/9*, and *NLRC5* expression as well as low *HLA-A* and *-C* expression (Figure 2C and Supplemental Figure 3E), consistent with the bulk RNA-Seq data. By contrast, cluster 6 (immune cells) displayed an average of 21-fold higher levels of *HLA-A*, *-B*, and *-C* transcripts.

Given the marked RNA- and protein-level downregulation of class I genes at baseline, we sought to identify a possible genetic basis for these observations. By whole-exome sequencing, no MCC lines harbored notable mutations in class I APM genes, except for *HLA-F* and *-H* mutations in MCC-320 (Supplemental Table 1). While 32 IFN pathway mutations were detected in all analyzed lines, only 2 were predicted as probably damaging by PolyPhen-2 (http://genetics.bwh.harvard.edu/pph2/), and no mutations were detected in *IFNGR1/2*, *JAK1/2*, *STAT1*, or *IRF1/2* (Supplemental Table 1). However, copy number loss of *NLRC5* was detected in 5 of 8 lines (62.5%) analyzed (Figure 2D and Supplemental Table 1). NLRC5 is a transcriptional activator that localizes to conserved S/X/Y regions within the promoters of class I pathway genes (33), and *NLRC5* copy number loss is a common alteration across many cancers (34). To determine whether *NLRC5* rescue was sufficient to restore surface HLA-I, we transfected vectors expressing *NLRC5* into IFN-γ–responsive lines (MCC-367) and nonresponsive lines (MCC-336, -350) (Supplemental Figure 3, F and G). However, increased *NLRC5* expression was not sufficient to appreciably increase surface HLA-I in any of the lines tested, suggesting additional downstream mechanisms of class I suppression.

### IFN-γ–induced HLA-I upregulation is associated with shifts in the HLA peptidome.

**Figure 3:**
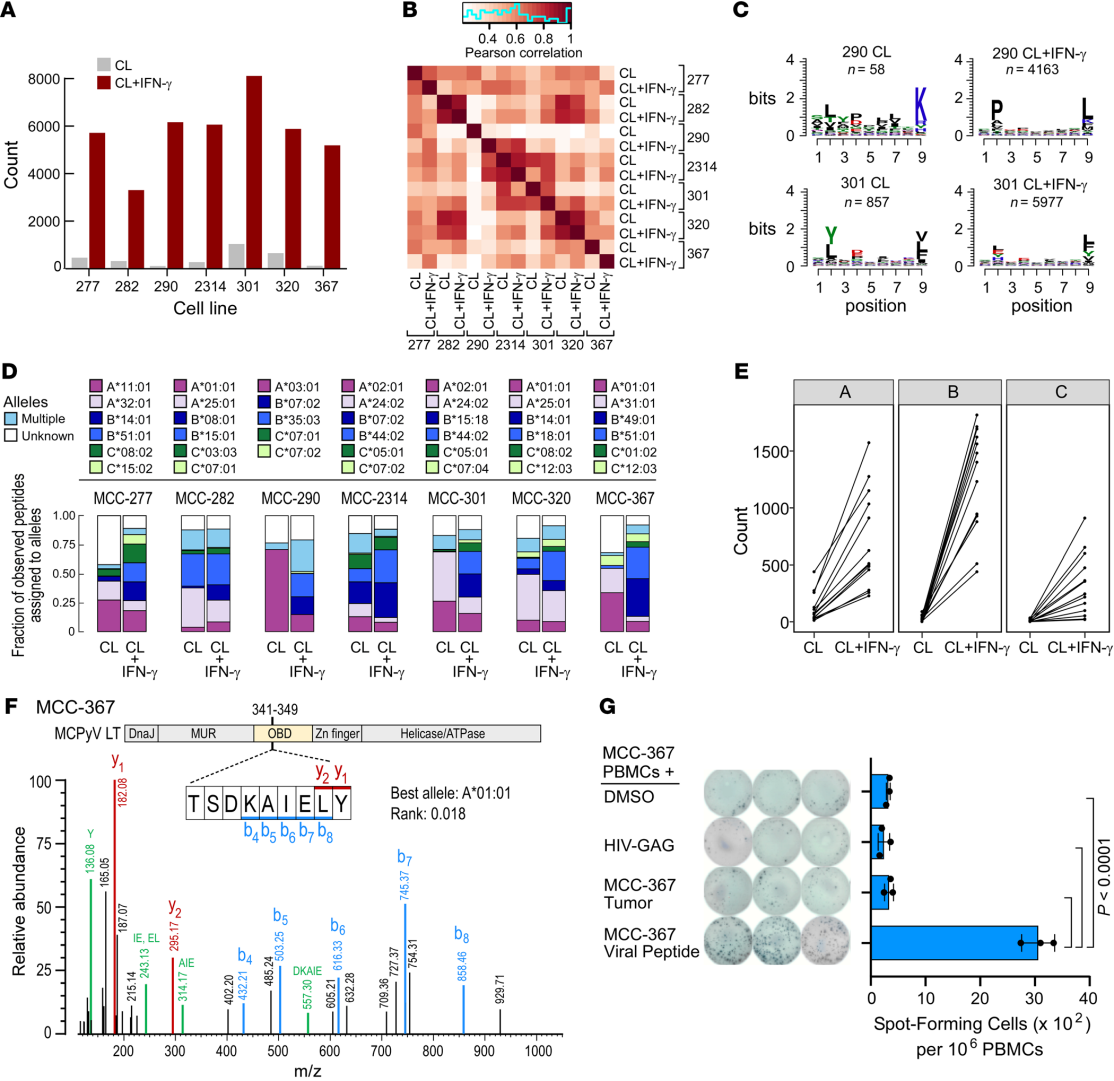
IFN-γ increases and alters the HLA peptidome in MCC. (**A**) Number of detected peptides presented on HLA-I in MCC lines at baseline (gray bars) and after IFN-γ treatment (red bars). CL, cell line. (**B**) Correlation heatmap of peptide sequences between MCC lines at baseline and after IFN-γ treatment in motif space. (**C**) 9-mer motif changes between untreated and IFN-γ–treated samples for MCC-290 (MCPyV^–^) and MCC-301 (MCPyV^+^) cell lines. (**D**) HLA allele distribution of presented peptides detected in cell lines at baseline and after IFN-γ treatment. Each HLA allele is represented by a different color. (**E**) Summary of changes in peptides presented per HLA gene upon IFN-γ treatment across all MCC lines analyzed for HLA-A (left), -B (middle), and -C (right). (**F**) Mass spectrum of a detected HLA-A–presented peptide derived from the MCPyV large T antigen (LT) in MCC-367. Red, blue, and green peaks represent y-, b-, and internal ions, respectively, confirming the peptide sequence. Internal ions are labeled with their respective amino acid sequences. MUR, Merkel cell virus T antigen unique region. OBD, origin-binding domain. (**G**) IFN-γ secretion by PBMCs from patient MCC-367 cocultured in an ELISPOT with DMSO, HIV-GAG negative control peptide, autologous MCC-367 tumor cells, or the LT-derived peptide identified in the MCC-367 HLA peptidome in **F**. Left: ELISPOT conditions. Right: Summary statistics (*n =* 3). *P* values were determined by 1-way ANOVA followed by post hoc Tukey’s multiple-comparison test.

Diminished expression of HLA-I would be expected to result in reduced numbers and diversity of HLA-presented peptides in MCC, impacting the immunogenicity of the tumor. Using our established workflows for direct detection of class I–bound peptides by liquid chromatography–tandem mass spectrometry (LC-MS/MS) (see Methods) (35), following immunoprecipitation of tumor cell lysates with a pan–HLA-I antibody (Supplemental Figure 4A), we detected low total peptide counts at baseline in parental tumors and cell lines (Supplemental Figure 4B). Following IFN-γ stimulation, we observed a median 12-fold increase in class I–bound peptide abundances across 7 cell lines, using comparable input material for immunoprecipitation (Figure 3A and Supplemental Figure 4B; and see Methods). The baseline immunopeptidome amino acid signature between the cell lines and parental tumors was highly correlated (Supplemental Figure 4C), and the cell line peptidomes shared more than 50% of their peptides with the corresponding tumor peptidomes (Supplemental Figure 4D). In contrast, we observed lower correlations before and after IFN-γ treatment and altered overall binding motifs with IFN-γ exposure (Figure 3, B and C, and Supplemental Figure 4E). To further explore these observations, we inferred the most likely HLA allele bound by the identified peptides. When comparing cell lines with and without IFN-γ treatment, we observed dramatic changes in the frequencies of peptides mapping to each HLA allele, most notably an increase in HLA-B–presented peptides (Figure 3, D and E). This is consistent with our previous observations that IFNs upregulate HLA-B more strongly than HLA-A (35), attributable to HLA-B having two IFN-responsive elements in its promoter (36, 37). Thus, the observed increase in HLA-B representation in IFN-γ–treated samples, and subsequent increase in HLA-B–presented epitopes, likely accounts for the aforementioned alterations in binding motifs.

For the MCPyV^+^ lines, we hypothesized that upregulation of HLA-I following IFN-γ stimulation would lead to increased ability to present MCPyV-specific epitopes. Indeed, for the MCPyV^+^ line MCC-367, we detected a peptide sequence derived from the origin-binding domain of LT antigen (TSDKAIELY), which was predicted as a strong binder for the HLA-A*01:01 allele present in that cell line (rank = 0.018, *HLAthena*) (ref. 35 and Figure 3F; and see Methods). This peptide was observed after IFN-γ treatment only in MCC-367. We subsequently confirmed reactivity against this MCC-367–derived epitope by autologous T cells by ELISPOT assay, demonstrating the immunogenicity of this epitope (Figure 3G).

### Complementary genome-scale gain- and loss-of-function screens to identify regulators of HLA-I in MCC.

**Figure 4:**
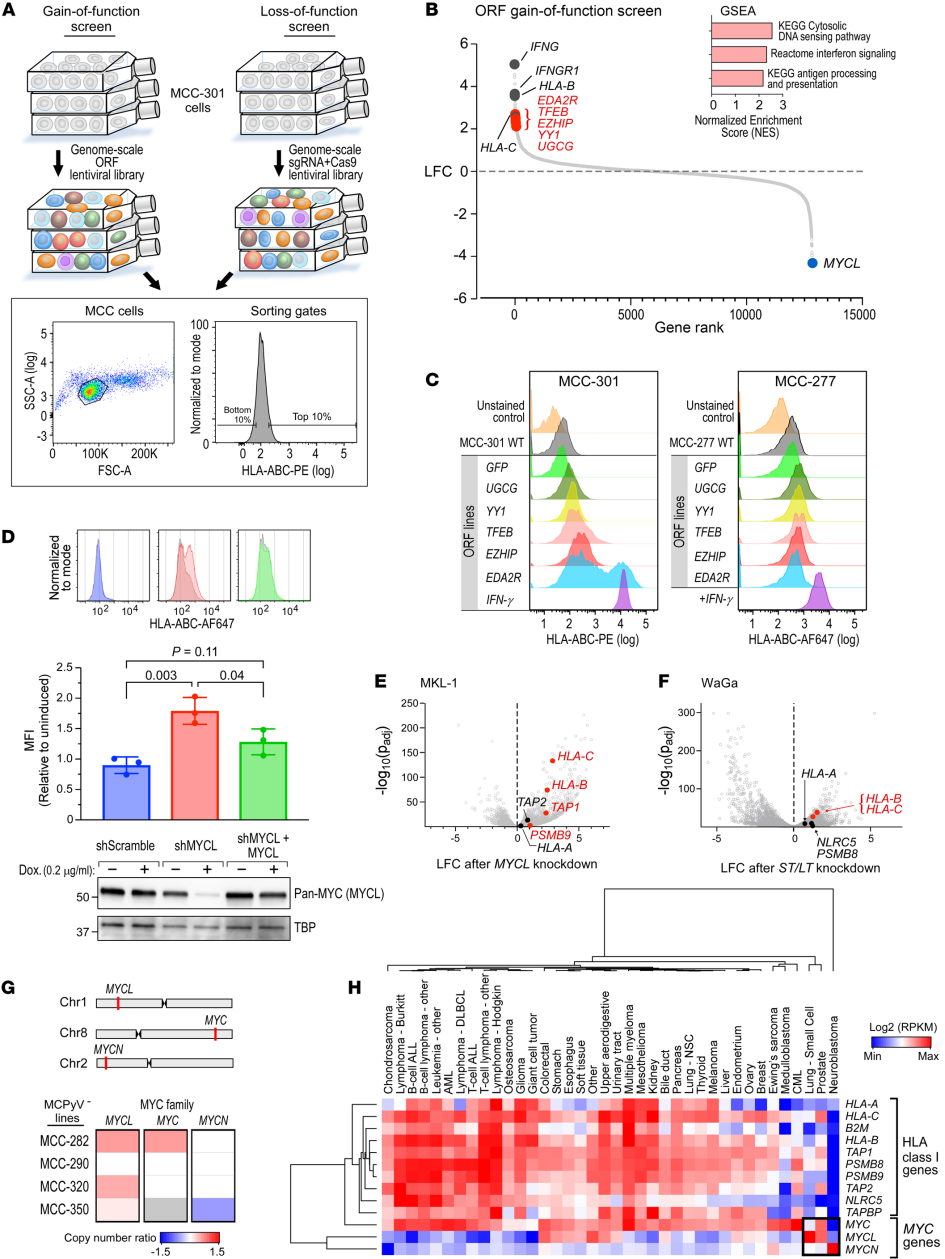
MYCL identified as a regulator of HLA-I through a genome-scale ORF screen. (**A**) Workflow and FACS gating strategy for the genome-scale ORF and CRISPR screens. (**B**) ORF screen results. Genes were ranked according to their log_2_(fold change) (LFC) enrichment in HLA-I–high versus –low populations. Inset: GSEA analysis of ORF positive hits. (**C**) HLA-I flow cytometry in MCC-301 (left) and MCC-277 (right) cells transduced with the indicated individual ORFs. Data visualized in log scale. (**D**) HLA-I flow cytometry in MKL-1 cells transduced with a doxycycline-inducible control shRNA, MYCL shRNA, or MYCL shRNA with rescue expression of MYCL. Top: Representative flow histograms. Middle: Normalized mean MFIs (*n =* 3). Bottom: Western blots for MYCL expression levels in each cell line. *P* values were determined by 1-way ANOVA followed by post hoc Tukey’s multiple-comparison test. Data visualized in log scale. (**E** and **F**) Volcano plots showing LFC expression in MKL-1 cells expressing shRNAs against *MYCL* (**E**) or in WaGa cells against both ST and LT (**F**), compared with control shRNA. Class I APM genes with *P*_adj_ < 0.05 and LFC > 1 are highlighted in red; other notable class I genes are in black. (**G**) Copy number variations in MYC family genes. Copy number gains and losses are shown in red and blue, respectively. Gray indicates no copy number variation data available. (**H**) Unsupervised clustering by Euclidian distance of RNA-Seq expression values of class I pathway genes and MYC family genes across all cancer cell lines in the Cancer Cell Line Encyclopedia (44). Median values displayed for each cancer type. ALL, acute lymphoblastic leukemia; AML, acute myeloid leukemia; CML, chronic myelogenous leukemia; DLBCL, diffuse large B cell lymphoma; NSC, non–small cell; RPKM, reads per kilobase per million mapped reads.

While *NLRC5* copy number loss was notable, the lack of HLA-I restoration with *NLRC5* overexpression and the simultaneous transcriptional downregulation of multiple class I genes suggested the presence of additional regulators. Thus, we performed paired genome-scale open reading frame (ORF) gain-of-function and CRISPR/Cas9-knockout (KO) loss-of-function screens in the MCPyV^+^ MCC-301 line to systematically identify regulators of HLA-I surface expression in MCC. We chose MCC-301 for several reasons. First, the low TMB of MCPyV^+^ MCC increases the likelihood of a shared mechanism for HLA-I suppression, which might relate to viral antigen signaling or cell type–specific factors. Second, IFN-γ–mediated inducibility of HLA-I largely excludes the possibility of hard-wired genomic alterations that would prohibit HLA-I upregulation. Last, such screens necessitate cell lines with robust growth such as MCC-301 (Supplemental Figure 1B). Thus, MCC-301 cells were transduced with ORF (38) or Cas9+sgRNA (39) lentiviral libraries in triplicate (see Methods). After staining of cells with an anti–HLA-I antibody, HLA-I–high and –low populations underwent FACS-based cell isolation (Figure 4A). Constructs were ranked according to their median log_2_(fold change) enrichment in the HLA-I–high versus –low populations, and for the CRISPR screen, sgRNA rankings were aggregated into gene-level rankings (39) (see Methods for analysis details).

### MYCL identified as a mediator of HLA-I suppression in MCC via ORF screen.

The ORF screen produced 75 hits with a >4-fold enrichment in HLA-I–high versus –low populations. As expected, these hits were highly enriched for IFN and HLA-I pathway genes by gene set enrichment analysis (GSEA) (ref. 40, Figure 4B, and Supplemental Table 2). The top hit was *IFNG*, with IFN pathway genes constituting 4 of the top 12 hits (33%). *HLA-B* and *-C* were ranked #10 and #38. Notably, transduction with the ORF library led to a population-wide increase in HLA-I, presumably due to IFN secretion from cells transduced with *IFN* gene ORFs. We confirmed this was an ORF library–specific effect and not due to lentiviral transduction, as GFP-transduced cells did not exhibit an increase in surface HLA-I (Supplemental Figure 5A). Furthermore, we confirmed that these notable hits exhibited high concordance between at least 2 replicates (Supplemental Figure 5, B and C).

We validated these positive hits by generating 71 single ORF overexpression lines in MCC-301, focusing on hits not related to IFN or HLA-I pathways. By flow cytometry, 8 of 71 candidate hits (11.3%) upregulated surface HLA-I more than 2-fold compared with GFP control while maintaining viability after transduction, including the Polycomb-related genes *EZHIP* (*CXorf67*) and *YY1* (Figure 4C). As further validation, we transduced these ORFs into the MCPyV^+^ MCC-277 line and confirmed increased levels of HLA-I (Figure 4C).

In contrast, *MYCL* was the top negative hit of the ORF screen (Figure 4B). MYCL is an important transcription factor in MCPyV^+^ MCC, as ST binds and recruits MYCL to the EP400 chromatin modifier complex to enact epigenetic changes necessary for oncogenesis (15, 41, 42). As validation, we observed that *MYCL* knockdown in MKL-1 cells increased surface HLA-I by flow cytometry (*P* = 0.003), an effect that was negated by rescue expression of exogenous *MCYL* (Figure 4D). To further investigate how MYCL affects HLA-I surface expression, we performed RNA-Seq of the MKL-1 *MYCL* shRNA line. Upon *MYCL* knockdown, we observed a more than 2-fold increase in expression of class I genes *HLA-B*, *HLA*-*C*, *TAP1*, and *PSMB9*, with enrichment for the signature of antigen processing/presentation by GSEA (*q* = 0.04) (Figure 4E, Supplemental Figure 5D, and Supplemental Table 3). Since ST binds and potentiates MYCL function through the ST-MYCL-EP400 complex (15), we suspected that viral antigen inactivation might also upregulate class I. To explore this, we transduced another established MCPyV^+^ MCC line, WaGa, with an shRNA targeting shared exons of *ST* and *LT*, leading to inactivation of both viral antigens. We observed a similar but more modest upregulation of class I genes, including >1.5-fold increases in *HLA-B*, *HLA-C*, and *NLRC5* (Figure 4F). Moreover, knockdown of *EP400* in MKL-1 with 2 different shRNAs resulted in a more than 3-fold increased level of *HLA-B* and *HLA-C* (Supplemental Figure 5E). These findings implicate the continued expression of ST-MYCL-EP400 complex components in the downregulation of HLA-I in MCC.

To explore the relationship between MYCL and HLA-I in MCPyV^–^ MCC and other cancers, we first evaluated the genomic status of *MYCL* in MCPyV^–^ MCC. Chromosome 1p copy number gain, encompassing *MYCL*, was previously reported as a common MCC copy number alteration (28, 43). Indeed, 3 of 4 (75%) of the MCPyV^–^ MCC lines exhibited *MYCL* copy number gain (copy number ratio 1.16–1.56; Figure 4G), suggesting a mechanism by which MCPyV^–^ MCC may enhance MYCL signaling in the absence of viral antigens. To determine whether *MYCL* is related to *HLA-I* expression in other cancers, we queried publicly available RNA-Seq data from the Cancer Cell Line Encyclopedia (44). Notably, other HLA-I–low neuroendocrine cancers such as small cell lung cancer and neuroblastoma featured overexpression of the MYC family proteins *MYCL* and *MYCN*, respectively (Figure 4H). Overall, *MYCL* exhibited negative correlation with average class I gene expression (Pearson’s correlation *r* = –0.33, *P* = 0.04).

### PRC1.1 complex identified as a negative regulator of HLA-I in MCC by CRISPR loss-of-function screen.

**Figure 5:**
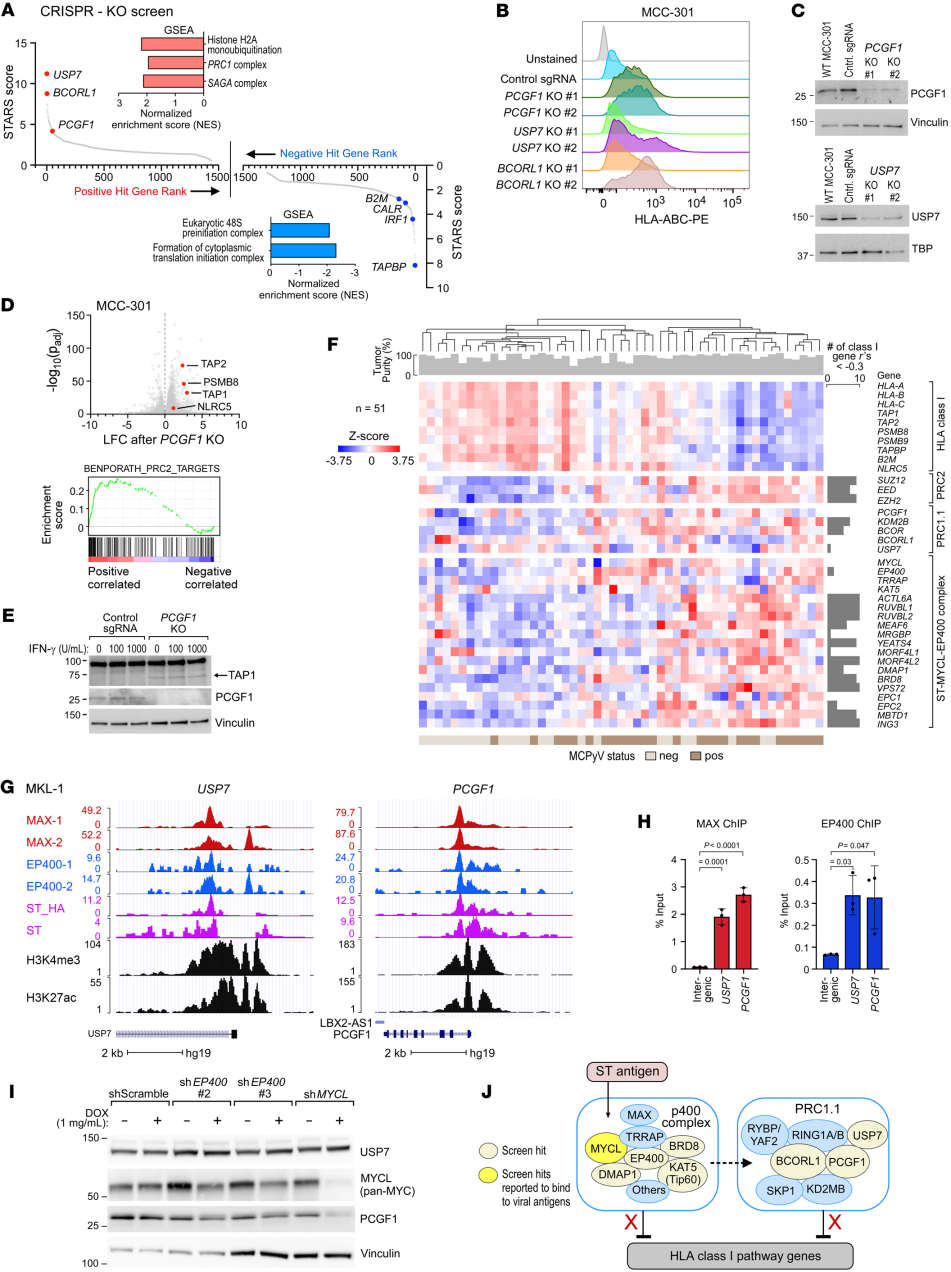
The PRC1.1 complex implicated as a suppressor of HLA-I in a genome-wide CRISPR screen. (**A**) Gene-level ranking of positive (left) and negative (right) CRISPR-KO screen hits, according to STARS, a gene-ranking algorithm for genetic screens (39). Inset: GSEA analysis of screen hits. (**B**) Flow cytometry for surface HLA-I in MCC-301 PRC1.1 KO lines (*PCGF1*, *USP7*, and *BCORL1*). Data visualized with biexponential scaling. (**C**) Western blot for PCGF1 (top) and USP7 (bottom) in WT MCC-301, a control sgRNA MCC-301 line, or the indicated knockout line. (**D**) Top: Volcano plot showing LFC in gene expression in an MCC-301 *PCGF1*-KO line compared with a control sgRNA line. Bottom: GSEA plot demonstrating enrichment of PRC2 target genes upon *PCGF1* knockout. (**E**) Western blot of TAP1 in *PCGF1*-KO and control sgRNA lines at varying IFN-γ concentrations. (**F**) RNA-Seq analysis of HLA-I genes, PRC1.1, PRC2, and ST-MYCL-EP400 in a cohort of 51 MCC tumors. Left: Unsupervised hierarchical clustering heatmap by Euclidian distance. Top track: Tumor purity scores for each tumor, generated by ESTIMATE (53). Pearson’s correlation coefficients between each PRC2, PRC1.1, or ST-MYCL-EP400 component and each class I gene were calculated, and the bar charts (right) show the number of Pearson’s coefficients that were less than –0.3. (**G**) UCSC Genome Browser view of *USP7* and *PCGF1* with ChIP-Seq tracks for MAX (red), EP400 (blue), MCPyV ST antigen (pink), and activating histone marks H3K4me3 and H3K27ac (black). The “-1” and “-2” suffixes refer to 2 different antibodies used for each protein. (**H**) ChIP-qPCR targeting the *USP7* and *PCGF1* promoters, using MKL-1 chromatin immunoprecipitated with a MAX (left) or EP400 (right) antibody (*n =* 3). *P* values were calculated by 1-way ANOVA followed by post hoc Dunnett’s multiple-comparison test. (**I**) Protein expression of USP7, PCGF1, and MYCL in MKL-1 cells transduced with the indicated doxycycline-inducible shRNAs. (**J**) Schematic of putative interactions between MCPyV viral antigens and screen hits MYCL and PRC1.1.

The CRISPR-KO screen also identified several class I genes. The top negative hit was *TAPBP* (Figure 5A and Supplemental Table 2), a chaperone that facilitates binding between unbound HLA-I and TAP (45). Other negative hits included the IFN pathway gene *IRF1* (#21) and class I genes *CALR* (#84) and *B2M* (#141). Having previously identified *MYCL* in our ORF screen, we observed other components of the ST-MYCL-EP400 complex among the CRISPR positive hits, including *BRD8* (#51), *DMAP1* (#93), *KAT5* (#619), and *EP400* (#886). Strikingly, we identified several components of the Polycomb repressive complex 1.1 (PRC1.1) among the positive hits, including the top 2 hits of the screen: *USP7* (#1), *BCORL1* (#2), and *PCGF1* (#50). For these genes, we observed high concordance between 2 CRISPR replicates (Supplemental Figure 6, A and B; and see Methods) and a more than 4.5-fold enrichment for at least 2 of the 4 sgRNAs (Supplemental Figure 6C).

PRC1.1 is a non-canonical Polycomb repressive complex that silences gene expression through mono-ubiquitination of H2AK119 in CpG islands. H2AK119ub facilitates recruitment of Polycomb repressive complex 2 (PRC2), which subsequently deposits repressive H3K27me3 marks (46). Other components of PRC1.1 include KDM2B, SKP1, RING1A/B, RYBP/YAF2, and BCOR (which can substitute for BCORL1; ref. 47). In aggregate, review of the top hits across the parallel screens revealed several hits related to Polycomb repressive complexes: PRC1.1 components *USP7*, *BCORL1*, and *PCGF1*; ORF hits *EZHIP* (which is an inhibitor of PRC2; ref. 48) and *YY1* (49); and PRC2 components *EED* and *SUZ12* (CRISPR positive hits #162 and #409).

We subsequently generated a series of MCC-301 KO lines against PRC1.1 genes *USP7*, *BCORL1*, and *PCGF1*. Knockout of each gene increased surface HLA-I by flow cytometry, most notably in the *PCGF1*-KO line (Figure 5B). *PCGF1* knockout also increased IFN-γ–induced HLA-I upregulation (Supplemental Figure 6D). Gene editing and protein knockout were confirmed by Sanger sequencing using tracking of indels by decomposition (TIDE) (ref. 50 and Supplemental Figure 6E) and by Western blot (Figure 5C), in genes for which antibodies were available.

To define the specific class I gene changes associated with PRC1.1 loss, we generated RNA-Seq data from the MCC-301 *PCGF1*-KO line, since *PCGF1* is essential for PRC1.1 function (51). Genes upregulated by *PCGF1* knockout were significantly enriched for the “PRC2 target genes” signature (Figure 5D), consistent with the known role of PRC1.1 in coordinating with PRC2 to repress target genes. *PCGF1* knockout caused a more than 5-fold increase in expression of class I genes *TAP1*, *PSMB8*, and *TAP2*, with a modest increase in *NLRC5* (Figure 5D). Furthermore, we observed increased protein expression of TAP1 by Western blot both at baseline and after IFN-γ treatment in the *PCGF1*-KO line (Figure 5E). Given the close relationship between PRC1.1 and PRC2, we next generated RNA-Seq and histone profiling data on MKL-1 cells treated with an inhibitor of the PRC2 member EZH2, with the hypothesis that PRC2 inhibition should mimic PRC1.1 knockout. Indeed, we observed similar upregulation of *TAP1* and *PSMB8* (Supplemental Figure 6G), with loss of repressive H3K27me3 in these genes’ promoters (ref. 52 and Supplemental Figure 6H). We then evaluated an RNA-Seq cohort of 51 MCC tumor biopsies to examine the association between class I genes and the chromatin-modifying complexes (PRC1.1, PRC2, and ST-MYCL-EP400) implicated by our screen hits. To account for potential immune cell infiltration, which might confound measurement of bulk class I expression, we applied ESTIMATE (53) to calculate tumor purity (median 87% purity, range 41%–99%). In aggregate, we observed consistent negative correlations with HLA class I genes for PRC2 and ST-MYCL-EP400 components (Figure 5F). For PRC1.1, we observed consistent negative correlations for *BCOR* and *KDM2B* (*P* < 0.05).

To explore the possible relationship between MYCL and PRC1.1, we reanalyzed previously generated ChIP-Seq data in MKL-1 cells (15). We observed that the ST-MYCL-EP400 complex members MAX and EP400 were bound to the promoters of the PRC1.1 genes *USP7* and *PCGF1*, but not *BCOR*/*BCORL1* (Figure 5G and Supplemental Figure 7A), and confirmed this by ChIP–quantitative PCR (Figure 5H). To assess whether this promoter occupancy was biologically relevant, we performed shRNA knockdown of *MYCL* and *EP400* in MKL-1 cells. We observed a notable decrease in PCGF1 protein levels after *MYCL* knockdown with a slight decrease after *EP400* knockdown, while USP7 levels remained relatively unchanged (Figure 5I). With limited validation (*n =* 2), we observed this trend in corresponding quantitative reverse transcriptase PCR experiments (Supplemental Figure 7B). These results indicate that PRC1.1 may act downstream of MYCL, most noticeably through MYCL’s regulation of PCGF1. Taken together with *MYCL’*s direct interaction with the MCPyV ST viral antigen (15), our results suggest a model by which the ST antigen coordinates with MYCL and PRC1.1 to suppress HLA-I surface expression (Figure 5J).

### Pharmacologic inhibition of USP7 restores HLA-I in MCC.

**Figure 6:**
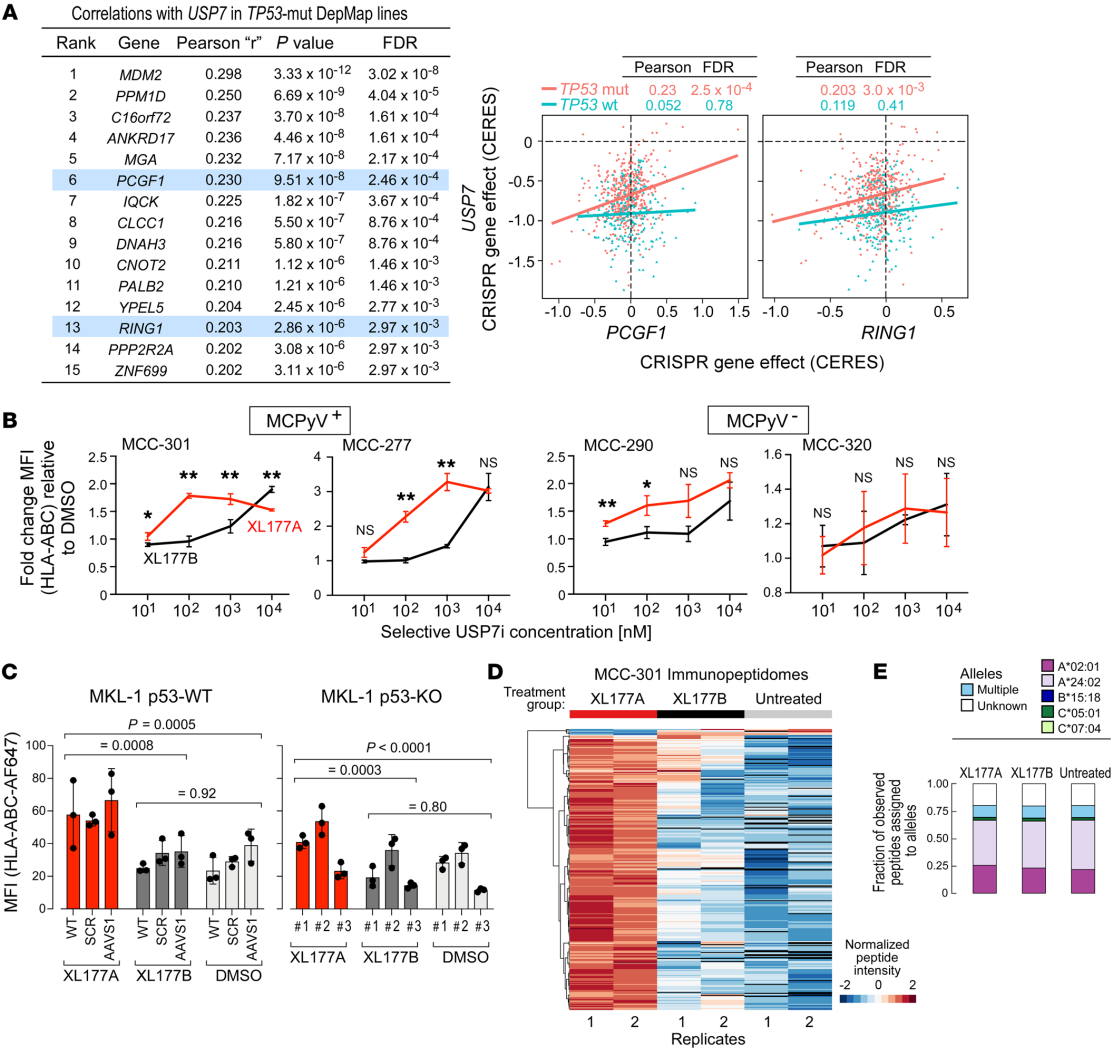
Pharmacologic inhibition of PRC1.1 component USP7 upregulates HLA-I in MCPyV^+^ MCC. (**A**) Dependency data from the Cancer Dependency Map (DepMap) (59, 60) were stratified based on *TP53* mutation status (*TP53*-mut [*n =* 532] vs. *TP53*-WT [*n =* 235]). Left: Pearson’s correlation coefficients with corresponding *P* values and FDRs of the top genes that are codependent with *USP7* in *TP53*-mutated lines, with PRC1.1 genes highlighted (see Supplemental Methods). Right: Graphical comparison of dependency of *USP7* with PRC1.1 genes *PCGF1* and *RING1* in *TP53*-WT (blue) and *TP53*-mut cell lines (red). The x- and y-axes display gene effect scores determined by CERES, an algorithm which estimates gene-dependency levels from CRISPR-Cas9 survival screens” (60) (**B**) Flow cytometry experiments measuring surface HLA-I in MCC lines treated with USP7 inhibitor XL177A or control compound XL177B, performed in technical triplicate. One-way ANOVA was performed, followed by Welch’s 2-tailed *t* tests comparing XL177A and XL177B MFIs, normalized to DMSO (see Methods). *P < 0.05; **P < 0.01; NS, *P* ≥ 0.05. (**C**) HLA I flow cytometry to assess the effect of USP7 inhibitors in MKL-1 p53-WT control lines (left) or p53-KO lines (right; lines 1–3 refer to 3 different single-cell p53-KO clones). Cells were treated with 100 nM XL177A (red), XL177B (black), or DMSO (light gray). For statistical analysis, 2-way ANOVA was performed, followed by post hoc Tukey’s multiple-comparison tests (see Methods). (**D**) Heatmap of peptide abundances within the HLA-I–presented peptidomes of MCC-301 cells treated with XL177A (red) or XL177B (black), compared with untreated cells (gray) (*n =* 2 replicates). Only peptides that were significantly differentially expressed between any 2 treatment groups (determined by 2-sample, 2-tailed *t* test) are shown. (**E**) Frequency of peptides presented on each HLA allele in MCC-301 cells treated with XL177A or XL177B, compared with untreated cells.

Selective small-molecule inhibitors of the PRC1.1 component USP7 have been previously developed (54, 55). However, since USP7 has many functions, such as regulation of p53 through MDM2 deubiquitination, and since its association with PRC1.1 was recently discovered (56–58), we queried the extent of USP7’s role in PRC1.1. By examining the Cancer Dependency Map (59, 60), we identified genes whose survival dependency correlated with that of *USP7* across cancer cell lines, with the rationale that survival codependency implies that such genes may function within the same complex or pathway. In contrast to *TP53*-wild-type (WT) lines, *TP53*-mutant lines showed a high correlation between *USP7* and PRC1.1 genes *PCGF1* and *RING1* (6th and 13th highest correlation coefficients, FDR = 2.46 × 10^–4^ and 2.97 × 10^–3^, respectively) (Figure 6A and Supplemental Table 4). Furthermore, GSEA revealed histone ubiquitination as the most enriched gene set within *USP7* codependent genes in *TP53*-mutant cell lines (Supplemental Figure 7C and Supplemental Table 4). These results support the notion that USP7 plays a role in PRC1.1 function.

We therefore assessed the activity of XL177A, a potent USP7 inhibitor, compared with XL177B, the corresponding enantiomer that is 500-fold less potent but exhibits on-target activity at higher doses (55). Two MCPyV^+^ lines (MCC-301, -277) and two MCPyV^–^ lines (MCC-290, -320) were treated for 3 days at varying inhibitor concentrations. At 100 nM, we observed a mean 2.0-fold (range 1.78–2.27) increase in expression of surface HLA-I by flow cytometry relative to DMSO in the MCPyV^+^ lines. Within the MCPyV^–^ lines, we noted a more modest increase in HLA-I in MCC-290 but not MCC-320 (Figure 6B). Given USP7’s prominent role in p53 regulation, we assessed whether USP7’s effect on HLA was p53 dependent. XL177A treatment of both *TP53*-KO and *TP53*-WT lines in MKL-1 increased surface HLA-I relative to XL177B and DMSO, albeit to varying degrees (Figure 6C and Supplemental Figure 7D). These results suggest that USP7 inhibitors exert some degree of p53-independent HLA-I upregulation, although we cannot rule out concurrent p53-dependent effects as well. Moreover, while USP7 inhibition did induce slight cell cycle shifts from S to G_1_ phase, this effect was similar in both *TP53-*WT and *TP53-*KO contexts (Supplemental Figure 7E). We then assessed the effect of USP7 inhibition in the MCC-301 *PCGF1*-KO line (Supplemental Figure 7F). We observed an increase in HLA-I surface expression in both control and *PCGF1*-KO contexts, indicative that USP7 inhibition may increase HLA-I through multiple mechanisms, not solely via PRC1.1. To evaluate the functional consequences of USP7 inhibition for HLA-I presentation, we analyzed the HLA-I–bound peptidomes of MCC-301 cells treated with XL177A and XL177B. XL177A-treated cells exhibited higher abundances of displayed peptides compared with XL177B-treated and untreated cells (Figure 6D and Supplemental Table 5). Of 282 peptides whose abundance significantly differed (*P* < 0.05) between 2 of the 3 conditions, 270 peptides (95.7%) were more abundant in XL177A-treated compared with untreated cells. Notably, XL177A treatment did not affect the frequency of peptides displayed on each respective HLA-I gene (HLA-A, -B, -C) (Figure 6E). This was consistent with our prior observation that *PCGF1* knockout mostly upregulated other class I genes related to peptide processing such as *TAP1/2* and *PSMB8*, rather than the *HLA-A*, *-B*, and *-C* genes themselves.

## Discussion

HLA-I loss is a widespread mechanism of immune evasion in cancer and facilitates resistance to immunotherapy (1–8). As a virally driven cancer, MCPyV^+^ MCC provides a highly informative substrate to study mechanisms by which viral antigens corrupt normal physiology. Just as the MCPyV LT antigen inactivates RB1 to phenocopy *RB1* mutations commonly seen in other cancers (14), we suspected that MCPyV viral antigens also suppress class I antigen presentation through derangement of regulatory mechanisms that might be phenocopied in other cancers, including MCPyV^–^ MCC tumors. Through unbiased genome-scale screens for regulators of HLA-I, we identified MYCL, which acts as part of the ST-MYCL-EP400 complex in MCPyV^+^ MCC and is frequently amplified in MCPyV^–^ MCC (15, 28, 43, 61). The ST antigen recruits MYCL to the EP400 complex to enact widespread epigenetic changes necessary for MCC oncogenesis, and our results identify an additional function of ST in suppressing HLA-I by MYCL activity. The effect of MYC family proteins on HLA generalizes to other cancers as well, as MYC and MYCN can suppress HLA-I in melanoma and neuroblastoma, respectively (62, 63).

The identification of PRC1.1 in our CRISPR screen highlights the importance of epigenetic regulatory mechanisms in suppressing HLA-I. PRC1.1 is a non-canonical Polycomb complex that mono-ubiquitinates H2AK119 within CpG islands, facilitating recruitment of PRC2, which deposits suppressive H3K27 trimethylation marks. PRC2 was recently identified as an HLA-I repressor through independent CRISPR screens in leukemia (64) and lymphoma cell lines (65), and this work demonstrates a connection to PRC1.1 as well. Those screens also identified *PCGF1* (Supplemental Table 2), while we identified PRC2 subunits in the CRISPR screen and the PRC2 inhibitor *EZHIP* (48) in the ORF screen. One limitation of our studies is the inability to rigorously validate all PRC1.1 KO lines, as *USP7* knockout resulted in substantial cellular toxicity and BCORL1-specific antibodies were not available to enable definitive confirmation of knockout. However, we observed that knockout of the essential PRC1.1 component *PCGF1* markedly upregulated *TAP2* and *PSMB8* (Figure 5D), and the similar expression changes seen with PRC2 inhibition provide additional validation (Supplemental Figure 6, G and H). Moreover, we observed a strong negative association between expression of PRC2 components and class I transcripts in an independent set of primary MCC samples (Figure 5F). Thus, our studies advance an emerging model in which cancers co-opt the Polycomb epigenetic machinery to suppress class I antigen presentation. Finally, we observed that the ST-MYCL-EP400 complex occupies the *PCGF1* promoter, with decreased PCGF1 protein levels after *MYCL* knockdown. This connection suggests a possible unifying mechanism by which MCPyV ST antigen co-opts MYCL to increase expression of PRC1.1, which subsequently suppresses class I gene expression in concert with PRC2.

Reversal of HLA-I loss is crucial for an effective antitumor cytotoxic T cell response. It is of high clinical interest that HLA-I–upregulating drugs could augment immunotherapy response. Our small-molecule USP7 inhibitor studies provide a promising avenue for pharmacologic upregulation of HLA-I in MCC. Given the diverse functions of USP7, future studies will be directed toward clarifying the degree to which PRC1.1 mediates these effects, as USP7 stabilizes numerous proteins, even MYC family proteins. We anticipate that continued in vitro and in vivo validation can pave the way for clinical use of USP7 inhibitors as an HLA-I–restoring adjunct.

## Methods

### Study design.

This study’s objective was to explore mechanisms of HLA-I downregulation and modulation in MCC, using 11 MCC cell lines we generated from frozen tumor biopsies or mouse PDXs. Informed patient consent was obtained under IRB protocol 09-156 at the Dana-Farber Cancer Institute (Table 1). No randomization was performed, and blinding was not relevant to this study. Experiments were performed in duplicate or triplicate. Means, standard deviations, and number of replicates are reported in the article. The definition and handling of outliers, when applicable, are described in the corresponding subsections of Methods and Supplemental Methods.

### MCC cell lines.

MCC tumor samples were obtained from patient biopsies or mouse PDXs, which were generated as previously described (66). The tissue was minced manually, suspended in 2 mg/mL collagenase I (Sigma-Aldrich), 2 mg/mL hyaluronidase (Sigma-Aldrich), and 25 μg/mL DNase I (Roche Life Sciences), and incubated on a low-speed orbital shaker for 30 minutes. After digestion, the single-cell suspension was passed through a 100 μm strainer, washed, and cultured in NeuroCult NS-A Human Proliferation Kit (Stemcell Technologies) supplemented with 0.02% heparin (Stemcell Technologies), 20 ng/mL human EGF (Miltenyi Biotec), and 20 ng/mL human FGF-2 (Miltenyi Biotec). Established cell lines were tested as mycoplasma free (Venor GeM Mycoplasma Detection Kit, Sigma-Aldrich). Cell lines were authenticated as MCC through IHC for CK20 and SOX2 (Figure 1A and Supplemental Figure 1C), and as derivatives of original tumors by HLA typing, which was available for 7 of the 11 lines (Supplemental Table 6). Cell line sexes are described in Table 1. MKL-1 and WaGa lines were gifts from James A. DeCaprio’s laboratory and were grown in RPMI 1640 with 10% FBS (Gibco) and 1% penicillin/streptomycin (Gibco).

### Flow cytometry.

Cells were dissociated with EDTA and incubated with 5 μL Human TruStain FcX (Fc Receptor Blocking Solution, BioLegend 422302) per million cells in 100 μL at room temperature for 10 minutes. Fluorophore-conjugated antibodies or respective isotype controls were added and incubated for 30 minutes at 4°C. Cells were washed once with PBS and resuspended in PBS or 4% paraformaldehyde and analyzed on an LSR Fortessa cytometer. For HLA-I and HLA-II detection, the following antibodies were used: HLA-ABC (W6/32 clone) conjugated to PE (BioLegend 311406), APC (BioLegend 311410), or Alexa Fluor 647 (AF647; Santa Cruz Biotechnology sc-32235 AF647), and HLA-DR–FITC (BioLegend 307604).

### Whole-exome sequencing and mutation calling.

Genomic DNA library preparation and next-generation Illumina whole-exome sequencing was performed by the Broad Institute Genomic Platform. Mutations were called using GATK v4.1.2.0 (67) with Mutect2 command (68). Called variants were filtered with the GATK FilterMutectCalls command (Supplemental Methods). Patient HLA allotype was assessed using standard class I and class II PCR-based typing (Brigham and Women’s Hospital Tissue Typing Laboratory).

### RNA-Seq analysis of MCC cell lines.

For samples from the MCC tumors and their derived cell lines, RNA-Seq libraries were prepared with Illumina’s TruSeq RNA Access Sample Prep Kit. Transcriptomes were sequenced on an Illumina flow cell to a coverage of at least 50 million reads in pairs. For fibroblast and keratinocyte control lines, raw FASTQ files were downloaded from the Sequence Read Archive using the R Bioconductor package SRAdb (69, 70) with accession codes SRP126422 (4 replicates from control samples “NN”) and SRP131347 (6 replicates with condition: control and genotype: control). For control MCC lines, raw FASTQ files of wild-type MKL-1 and the control WaGa line described in Supplemental Methods (see section *MKL-1 shMYCL and WaGa shST/LT cell line generation and RNA-Seq*) were used (doxycycline-inducible ST/LT shRNA, without doxycycline treatment). Analysis of RNA-Seq is subsequently described in Supplemental Methods.

### Immunoprecipitation, mass spectrometry analysis, and peptide identification.

Forty million or 0.2 g of MCC cells with or without IFN-γ treatment were immunoprecipitated and analyzed by LC-MS/MS (Supplemental Methods). Mass spectra were interpreted using Spectrum Mill software package v7.1 pre-release (Broad Institute) (refs. 35, 71, and Supplemental Methods).

Immunopeptidomes of USP7 inhibitor–treated cell lines were eluted similarly to the method described above, followed by labeling with TMT6 reagent (Thermo Fisher Scientific; XL177A-treated replicates labeled with TMT6-126 and -128; XL177B-treated replicates labeled with TMT6-130 and -131; and WT replicates labeled with TMT6-127 and -129), and then pooled for subsequent fractionation using basic reversed-phase fractionation with increasing concentrations of acetonitrile (10%, 15%, and 50%) in 5 mM ammonium formate (pH 10) and analysis on an Orbitrap Exploris 480 with FAIMS Pro (Thermo Fisher Scientific). Data acquisition parameters were as described with normalized collision energy set to 34% and dynamic exclusion set to 2 seconds.

### Whole-proteome analysis and interpretation.

Protein expression of MCC cell lines was assessed as described previously (72). Briefly, cell pellets of MCC cell lines with and without IFN-γ treatment were lysed in 8 M urea and digested to peptides using LysC and trypsin (Promega). Four hundred micrograms of peptides were labeled with TMT10 reagents (Thermo Fisher Scientific) and then pooled for subsequent fractionation and analysis. Pooled peptides were separated into 24 fractions using offline high-pH reversed-phase fractionation. One microgram per fraction was analyzed by LC-MS/MS, and data were analyzed using Spectrum Mill (Supplemental Methods).

### ELISPOT.

MCC-367 PBMCs were stimulated with 10 μg/mL of the LT antigen peptide TSDKAIELY (identified in the MCC-367 HLA peptidome; Figure 3F) in DMEM supplemented with 10% human serum and 20 ng/mL IL-7 (PeproTech). After 3 days, cells were supplemented with 20 U/mL IL-2 (PeproTech). After 10 days, cells were cytokine deprived overnight. Fifty thousand cells per well were stimulated in an IFN-γ ELISPOT assay with 10 μg/mL of the TSDKAIELY peptide (negative controls, DMSO, HIV-GAG; positive controls, CEF [Mabtech], PHA [Sigma-Aldrich]). ELISPOT and T cell culture methods were described in detail previously (23, 73).

### ORF screen.

The human ORFeome version 8.1 lentiviral library (38) was a gift from the Broad Institute Genetic Perturbation Platform. Seventy-five million MCC-301 cells were transduced with ORFeome lentivirus to achieve an infection rate of 30%–40%. Two days later, transduced cells were selected with 3 days of 0.5 μg/mL puromycin (Santa Cruz Biotechnology sc-10871) treatment. Between 7 and 10 days after transduction, cells were stained with an anti–HLA-ABC–PE antibody (W6/32 clone, BioLegend 311405) and sorted on a BD FACSAria II, gating for the top and bottom 10% of HLA-ABC–PE staining. Sorted cells were washed with PBS, flash-frozen, and stored at –80°C. Genomic DNA was isolated, followed by indexed PCR amplification of the construct barcode and sequencing on an Illumina HiSeq. The screen was performed in triplicate. Screen data analysis is described in Supplemental Methods.

### CRISPR-KO screen.

The Brunello human CRISPR knockout pooled plasmid library (39) (1-vector system) was a gift from David Root and John Doench (Addgene 73179). The plasmid library was amplified in ElectroMAX *Stbl4* Competent cells (Thermo Fisher Scientific 11635018), and maintenance of library diversity was confirmed by Illumina sequencing of PCR-amplified sgRNA barcode regions. To produce lentivirus, HEK293T cells were transfected with Brunello plasmid, VSV-G, and psPAX2 plasmids using TransIT-LT1 (Mirus MIR2300). Lentivirus was harvested 48 hours after transfection and flash-frozen. Lentiviral transduction and FACS screening were performed in triplicate analogously to the ORF screen with the following exceptions: 150 million MCC-301 cells were transduced per replicate, cells were sorted 10–14 days after transduction, representative pellets (40 million cells) after transduction but before flow cytometry selection were sequenced to assess sgRNA representation (Supplemental Figure 6A), and 1 replicate was excluded from analysis because of poor sample quality. Screen data analysis and exclusion criteria are described in Supplemental Methods.

### Generation of ORF lines.

Single ORF constructs cloned into the pLX_TRC317 plasmid were a gift from the Broad Institute Genetic Perturbation Platform. ORF plasmids, psPAX2, and VSV-G were transfected into HEK293T cells to produce lentivirus. MCC-301 and MCC-277 cells were transduced with individual ORF lentivirus in 2 μg/mL Polybrene, and spinfection was performed at 532 *g* for 2 hours at 30°C. Two days after transduction, transduced cells were selected with 3 days of 0.5 μg/mL puromycin treatment.

### Generation of CRISPR-KO lines.

Forward and reverse oligonucleotides with the sequence 5′-CACCG---[sgRNA sequence]---3′ and 5′-AAAC---[reverse complement of sgRNA]---C-3′ were synthesized by Eton Biosciences, then annealed and phosphorylated, producing BsmBI-compatible overhangs. LentiCRISPRv2 vector (Addgene 52961) was digested with BsmBI, dephosphorylated with shrimp alkaline phosphatase, and gel-purified. Vector and insert were ligated at a 1:8 ratio with T7 DNA ligase at room temperature and transformed into *Stbl3* chemically competent cells (Thermo Fisher Scientific C737303). Cloning was confirmed via Sanger sequencing using the primer 5′-GATACAAGGCTGTTAGAGAGATAATT-3′. Lentivirus was produced in HEK293T cells (psPAX2, VSV-G, and cloned CRISPR plasmid), and MCC-301 cells were transduced with single-construct lentivirus for single-knockout lines, or with 2 lentivirus pools containing 2 different sgRNAs against the same gene for double-knockout lines. Transduction was performed in the same manner as for the CRISPR-KO library. Gene editing was confirmed by TIDE (50).

### Western blot analysis.

Western blots were run in 4%–20% gradient gels (Bio-Rad). Primary antibodies used include USP7 (Life Technologies PA534911), PCGF1 (E8, Santa Cruz Biotechnology sc-515371), TAP1 (Cell Signaling Technology 12341S), TAP2 (Cell Signaling Technology 12259S), p53 (Santa Cruz Biotechnology sc-126), pan-MYC (Abcam ab195207), vinculin (Sigma-Aldrich V9131), and TBP (Cell Signaling Technology 8515S), diluted according to the manufacturers’ specifications. Secondary antibodies used include Bethyl Laboratories goat anti-mouse (A90-116P) and goat anti-rabbit (A120-101P). Blots were incubated in Immobilon Western Chemiluminescent (Millipore) HRP substrate and visualized on the G-box imaging system (Syngene). Raw Western blot images were processed for visualization using ImageJ software (NIH). Full uncut gels are available in the Supplemental Materials.

### MKL-1 shMYCL and WaGa shST/LT RNA-Seq and flow cytometry.

MKL-1 shMYCL and WaGa shST/LT cell line generation and RNA-Seq (15) are described in detail in Supplemental Methods. For GSEA, genes were ranked based on their log_2_(fold change) value from DESeq2 (Bioconductor). These ranked lists were then used as input for GSEAPreranked (enrichment statistic, weighted; maximum gene set size, 500; minimum gene set size, 15). For flow cytometry, shMYCL and shScr MKL-1 cells were treated with 0.2 μg/mL doxycycline for 7 days before flow cytometry. In addition, shMYCL cells containing a constitutively expressed (Addgene 17486) shRNA-resistant MYCL (shMYCL+MYCL) construct were treated identically to control for any off-target effects of the shRNA.

### PCGF1-KO RNA-Seq.

RNA-Seq was performed on 3 technical replicates from the MCC-301 *PCGF1*-KO #2 line and MCC-301 transduced with a nontargeting sgRNA (see above, section *RNA-Seq analysis of MCC cell lines*). FASTQ files were assessed for sequencing quality via FastQC (Babraham Institute), with those of passing quality used for further analysis. Salmon (74) was used to map raw reads to the decoy-aware transcriptome of GRCh38p.13 v99 (Ensembl) with the following stipulations: --writeUnmappedNames, --seqBias, --gcBias, --validateMappings. Raw transcript-level counts were converted to gene-level counts via TxImport (75), and differential gene expression analysis was performed using DESeq2 (76).

### ChIP-Seq and ChIP-qPCR.

ChIP-Seq data for MAX, EP400, ST, H3K4me3, and H3K27ac were generated as previously described (15). The ChIP–quantitative PCR (qPCR) primers, shown in Supplemental Table 7, were designed using PrimerQuest (IdtDNA) based on ChIP-Seq data displayed in the UCSC Genome Browser. qPCR was performed using the Brilliant III Ultra-Fast SYBR Green qPCR Master Mix (Agilent) on the AriaMx Real-time PCR System (Agilent) following the manufacturer’s instructions.

### Western blot and qPCR analysis of shMYCL and shEP400 in MKL-1 cells.

MKL-1 cells transduced with doxycycline-inducible shRNA targeting MYCL (shMYCL), EP400 (shEP400 #2, #3), or a nontargeting control (shScr) were treated with 1 μg/mL doxycycline for 5 days, with retreatment of fresh doxycycline-containing media on day 3. Western blots were performed as described above. For qPCR analysis, cDNA was quantified on the AriaMx (Agilent) using Brilliant III Ultra-Fast qPCR Master Mix (Agilent). Raw Ct values were normalized to 18S rRNA via the ΔΔCt method. Primers are listed in Supplemental Table 7.

### USP7 inhibitor experiments in wild-type MCC.

MCC cells (2.5 million) were incubated with USP7 inhibitor XL177A or control enantiomer XL177B at 10 μM, 1 μM, 100 nM, and 10 nM for 3–4 days. HLA-I flow cytometry was performed as described above. MCC-301 data are representative of 4 independent experiments. For each line, 1-way ANOVA was performed on the MFIs of the DMSO group and all experimental groups. Individual Welch’s 2-tailed *t* tests were performed for each concentration, comparing the fold changes of MFI (inhibitor) / mean MFI (DMSO control) between XL177A and XL177B.

### USP7 inhibitor experiments in p53-KO lines.

p53 knockout and control lines (WT, scrambled, AAVS1) were generated in MKL-1 cells (77) with USP7 inhibitors and subjected to HLA-I flow cytometry. Because the root mean squared error differed considerably between the control lines and the p53-KO lines (12.2894 and 6.69844), the 2 groups were analyzed separately by 2-way ANOVAs, and drug treatment was found to be a statistically significant source of variation in MFI in both cases (*P* = 0.0003 in controls and *P* < 0.0001 in p53-KO lines). ANOVA was followed by post hoc Tukey’s multiple-comparison tests between XL177A, XL177B, and DMSO treatments to generate the *P* values displayed in Figure 6C.

### Data and material availability.

All MCC lines generated in this study are available upon request. Data from whole-exome sequencing, RNA-Seq of MCC cell lines and parental tumors, scRNA-Seq, whole-genome sequencing are available in the Database of Genotypes and Phenotypes (dbGaP phs002260). All analysis code for WES analysis, RNA-Seq analysis, MCPyV viral transcript detection is available in a GitHub repository under an MIT license at github.com/kdkorthauer/MCC (Commit ID 1f369fd). The original mass spectra for all proteomics and immunopeptidomics experiments, tables of peptide spectrum matches for immunopeptidome experiments, and the protein sequence databases used for searches have been deposited in the public proteomics repository MassIVE (https://massive.ucsd.edu) and are accessible at ftp://MSV000087251@massive.ucsd.edu (username, MSV000087251; password, modulation).

### Statistics.

Flow cytometry bar graphs show MFI of 3 technical or biological replicates, except for Figure 1D and Supplemental Figure 2C, which show 1 sample. Error bars indicate SD, unless otherwise stated. Bar charts without displayed data points contain only 1 data point per column. A *P* value of 0.05 was used as the significance threshold. Specific statistical tests, analyses, and software are described in the figure legends and/or Methods. No randomization procedures or sample size calculations were carried out as part of the study. Analysis code for sequencing data is available in a GitHub repository under an MIT license at www.github.com/kdkorthauer/MCC All analyses in R were carried out using version 3.6.2.

### Study approval.

Informed consent was obtained from MCC patients prior to inclusion in this study, which was approved by the IRB (protocol 09-156) at the Dana-Farber Cancer Institute. For mouse studies, PDX generation was approved by the Dana-Farber Cancer Institute IACUC (66).

## Author contributions

PCL, SK, PML, JC, VA, TCF, JDS, AYLW, JBI, AYT, VAC, IPC, AGK, SS, KTW, LWL, SL, TH, LP, AMA, LJW, SR, KDF, JOW, EW, WZ, and DBK performed experiments. SK, KK, TCF, CKL, CBP, LMD, JN, DN, and GJS analyzed data. XL, SJB, and MT provided samples and reagents. LRO, SJR, KRC, WJL, GZ, JGD, SAC, JAD, CJW, and DBK supervised experiments. PCL, SK, JAD, CJW, and DBK wrote the manuscript.

## Supplementary Material

Supplemental data

Supplemental table 1

Supplemental table 2

Supplemental table 3

Supplemental table 4

Supplemental table 5

Supplemental tables 6-7

## Figures and Tables

**Table 1:**
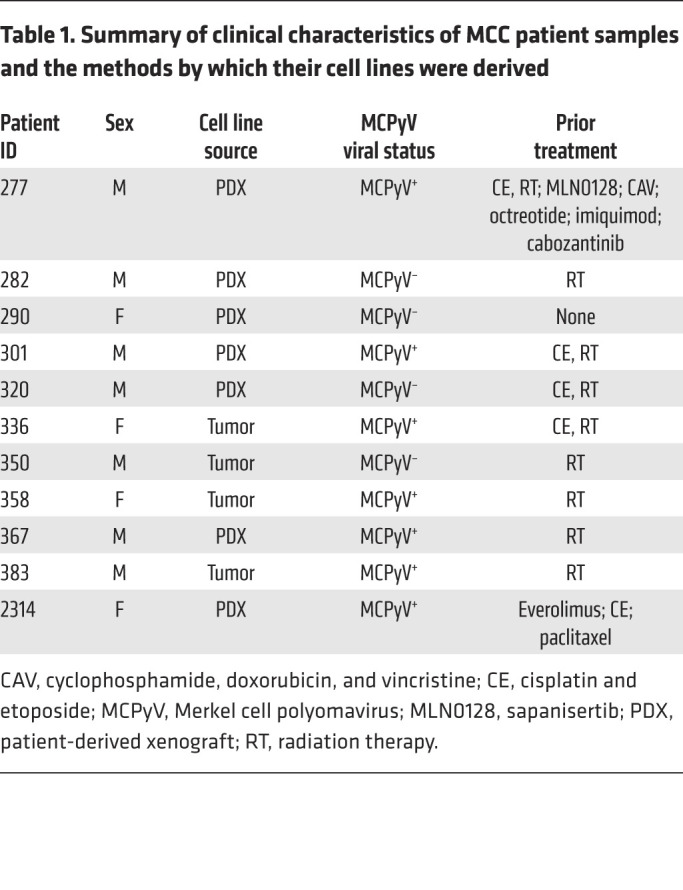
Summary of clinical characteristics of MCC patient samples and the methods by which their cell lines were derived

## Version 1 07/01/2022

Electronic publication
