# Supplementary material for: Reversal of viral and epigenetic HLA class I repression in Merkel cell carcinoma
Source: J Clin Invest. 2022 Jul 1;132(13):e151666. doi: 10.1172/JCI151666 (PMC9246387; doi:10.1172/JCI151666)
Supplement: Supplemental data [file jci-132-151666-s194.pdf]

Supplemental Data

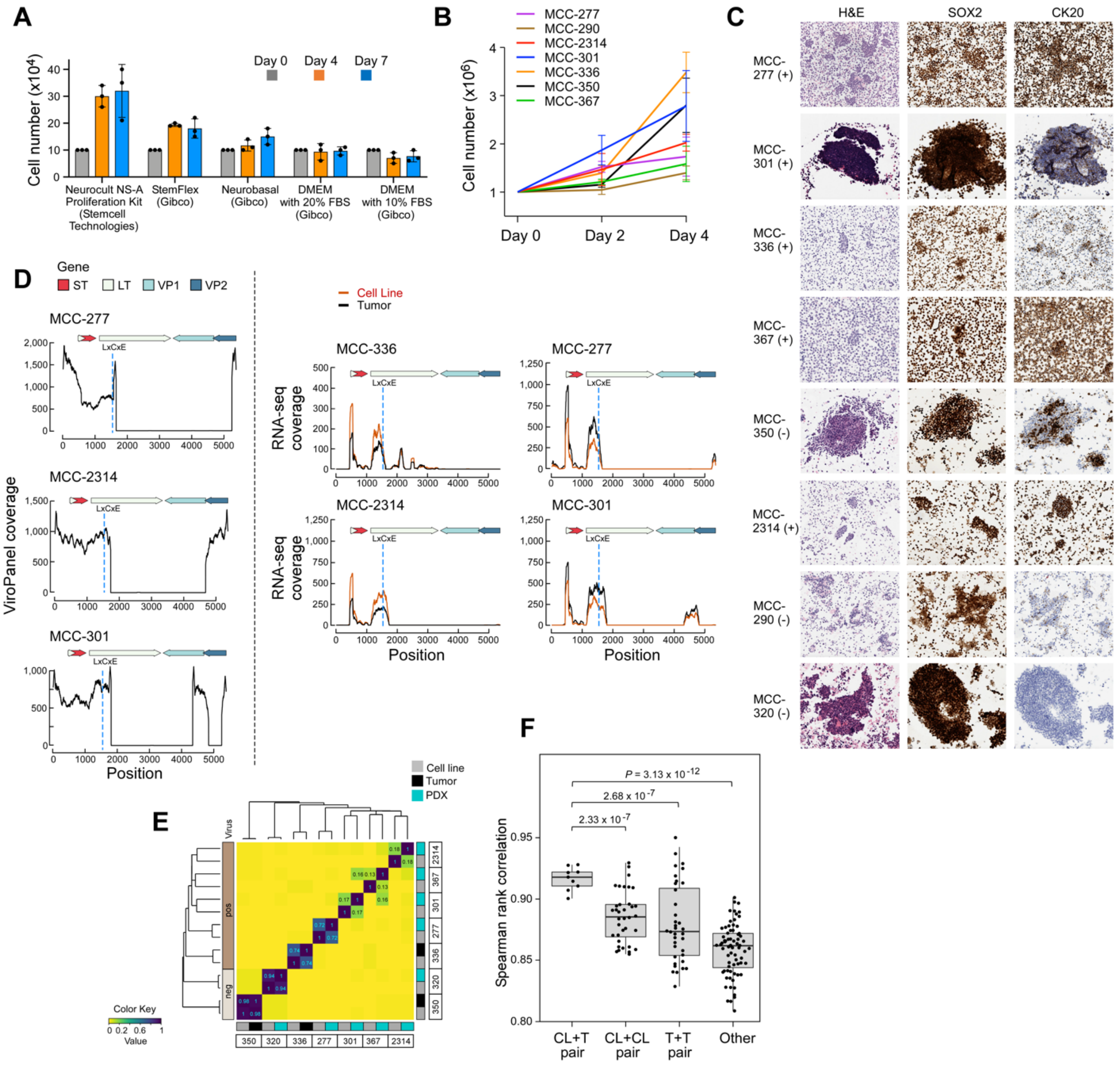

Supplemental Figure 1. Further characterization of 11 novel MCC cell lines. A) Cell culture media optimization in the MCC-336 cell line. Cells were counted at day 0, 4, and 7 (n=3 replicates derived from original tumor). **B)** Growth curves of newly generated MCC cell lines. One million cells were seeded in triplicate on Day 0 and counted at Day 2 and Day 4. **C)** Immunohistochemistry for 8 of the newly generated MCC cell lines, with staining for MCC

markers SOX2 and CK20 (20x magnification). Images for MCC-277 and -350 were previously shown in **Figure 1A**. **D)** MCPyV genome coverage at the DNA level detected by ViroPanel (left) and at the transcriptional level detected by RNA-seq (right). **E)** Clustering of MCC tumors and cell lines by similarity in mutational profiles. Similarity scores were calculated based on the concordant presence or absence of mutations between tumor and cell line on a 0 to 1 scale, where a score of 1 indicates identical profiles. **F**) Pairwise Spearman correlations based on RNA-seq data for corresponding tumor-cell line pairs, along with all possible tumor-tumor pairs, cell line-cell line pairs, and all other pairings. Center line, median; box limits, upper and lower quartiles; whiskers, range excluding outliers.

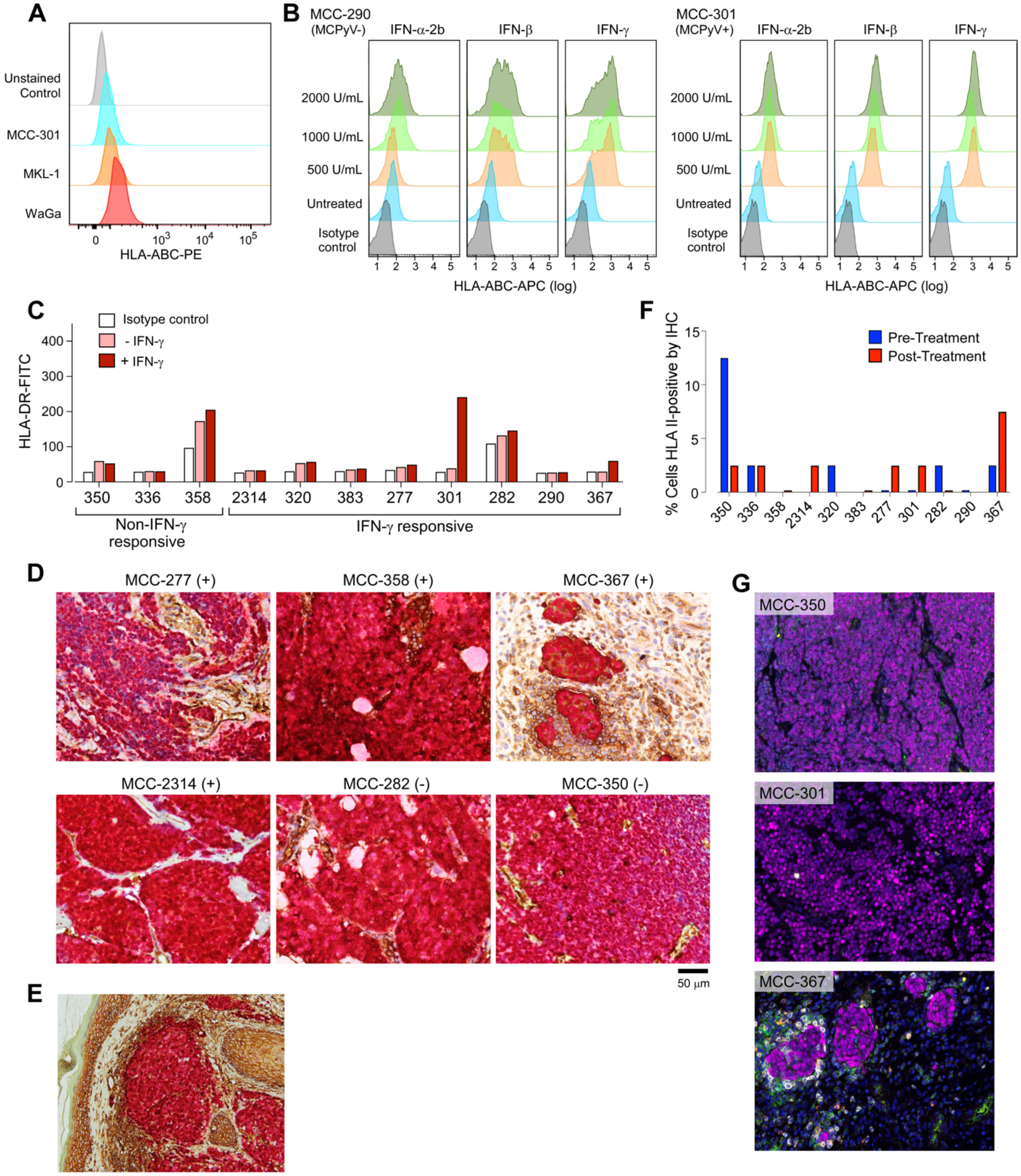

Supplemental Figure 2. Effects of interferons on HLA-I and -II expression in MCC lines and IHC characterization. (**A**) Flow cytometry experiments measuring HLA-I surface expression (W6/32 antibody, PE) in two established MCPyV+ lines, MKL-1 and WaGa,

alongside MCC-301. Data visualized with biexponential scaling. (**B**) Effect of type I and type II IFNs on surface MHC I expression in MCC by flow cytometry. 5 x10^5 MCC cells were treated with the indicated doses of IFNα2b, IFNB1, or IFNG for 24 hours. Representative histogram plots show cells stained with anti-HLA-I (W6/32, APC) or isotype antibodies. The experiment was performed in the MCPyV- line MCC-290 (left) and the MCPyV+ line MCC-301 (right). Data visualized in log scale. (**C**) Flow cytometry assessment of HLA-DR expression in all 11 MCC lines, both at baseline (light pink) and after IFNG treatment (red), compared to isotype control (white). Categorization of non-IFNG-responsive versus IFNG-responsive is based upon HLA class I, as shown in **Figure 1D**. (**D**) IHC images of parental MCC tumors, stained for HLA class I (brown) with SOX2 co-stain (red) to identify MCC cells. An additional image for MCC-282 (**E**) that more prominently features SOX2-negative epidermal keratinocytes is provided to highlight specificity of SOX2 staining for MCC cells (20x magnification). (**F)** Summary of the percent of MCC cells that are HLA II-positive within available pre- (n=6) and post-treatment (n=9) tumor samples (see **Table 1** for prior treatments). MCC cell lines were derived from post-treatment samples. (**G**) Representative multiplex immunofluorescence images of MCC FFPE tumor tissue sections. Probes include DAPI nuclear (blue), CD8 (white), FOXP3 (yellow), PD-1 (orange), PD-L1 (green), and SOX2 (magenta).

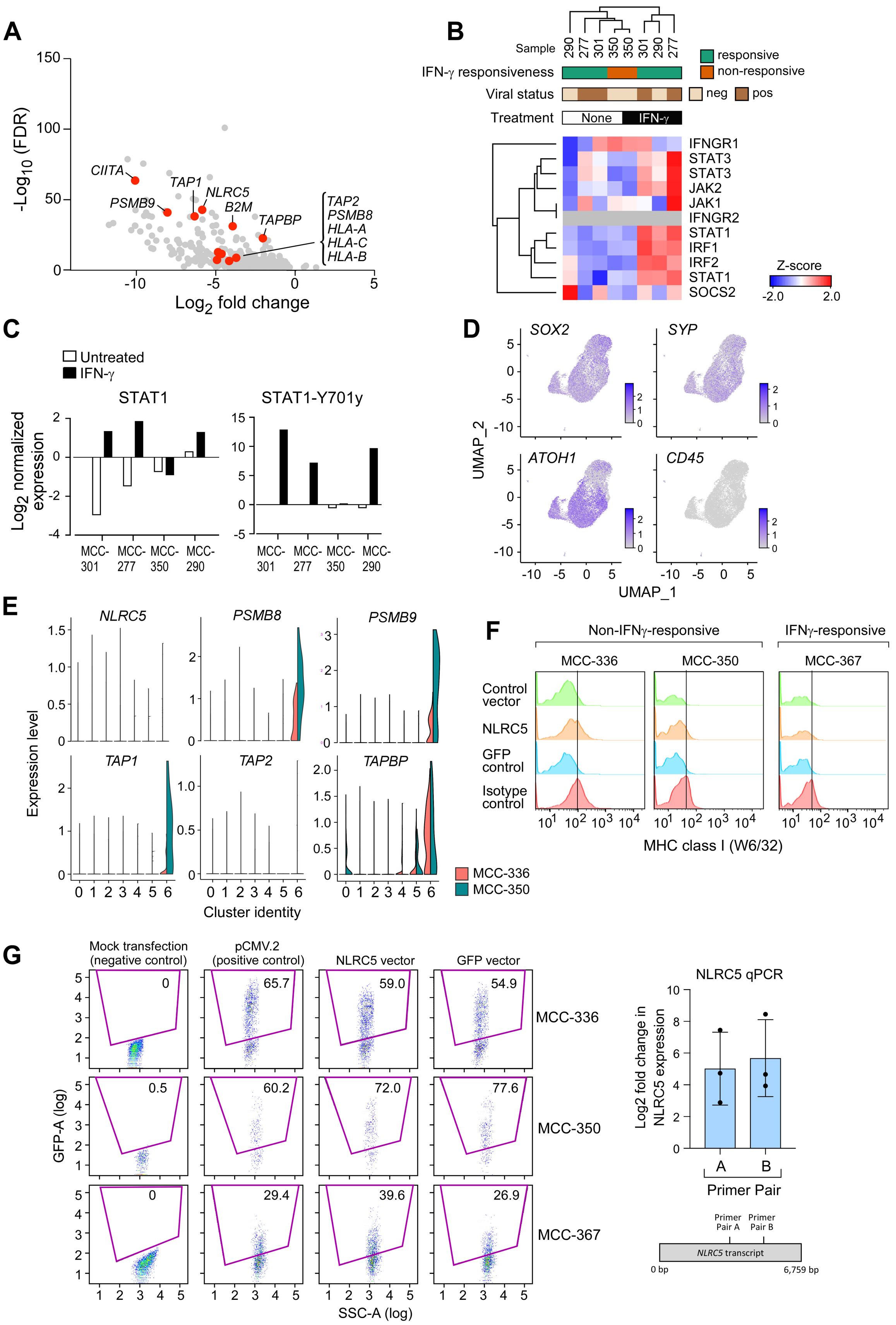
A
CIITA
PSMB9
TAP1
NLRC5
B2M
TAPBP
TAP2
PSMB8
HLA-A
HLA-C
HLA-B
-Log10 (FDR)
Log2 fold change
B
Sample
290 277 301 350 350 301 290 277
IFN-γ responsiveness
responsive
non-responsive
Viral status
neg
pos
Treatment
None
IFN-γ
IFNGR1
STAT3
STAT3
JAK2
JAK1
IFNGR2
STAT1
IRF1
IRF2
STAT1
SOCS2
Z-score
-2.0
2.0
C
Untreated
IFN-γ
STAT1
STAT1-Y701y
Log2 normalized expression
MCC-301
MCC-277
MCC-350
MCC-290
D
SOX2
SYP
ATOH1
CD45
UMAP_2
UMAP_1
E
NLRC5
PSMB8
PSMB9
TAP1
TAP2
TAPBP
Expression level
Cluster identity
MCC-336
MCC-350
F
Non-IFNγ-responsive
IFNγ-responsive
MCC-336
MCC-350
MCC-367
Control vector
NLRC5
GFP control
Isotype control
MHC class I (W6/32)
G
Mock transfection (negative control)
pCMV.2 (positive control)
NLRC5 vector
GFP vector
0
65.7
59.0
54.9
MCC-336
0.5
60.2
72.0
77.6
MCC-350
0
29.4
39.6
26.9
MCC-367
GFP-A (log)
SSC-A (log)
NLRC5 qPCR
Log2 fold change in NLRC5 expression
A
B
Primer Pair
Primer Pair A
Primer Pair B
NLRC5 transcript
0 bp
6,759 bp

Supplemental Figure 3. MCC lines exhibit low HLA-I expression at both the bulk and single cell level. (**A**) Volcano plot of differentially expressed genes with FDR < 0.01 (notable HLA-I genes shown in red) between baseline and IFNG-treated MCC cell lines. Negative LFC indicates increased expression in +IFNG samples. (**B**) Proteomics heatmap depicting the relative expression of key IFNG pathway components in 4 MCC lines, both at baseline and after IFNG treatment. Gray shading indicates that the protein was not detected. (**C**) Targeted analysis of normalized STAT1 peptide counts (left) and STAT-Y701y phosphosite counts (right) between untreated and IFNG-treated cell lines. Absence of bar indicates that the peptide/phosphosite was not detected in that particular sample. (**D**) scRNA-seq expression of MCC markers *SOX2*, *ATOH1*, and synaptophysin (*SYP*), and immune cell marker *CD45* within the MCC-336 and -350 tumor samples. (**E**) scRNA-seq expression of additional HLA-I genes across all clusters (clusters 0-5: MCC; cluster 6: immune cells). **(F)** Flow cytometry of surface HLA-I expression in *NLRC5*-overexpressing MCC lines. Both IFNG- responsive (MCC-367) and non-IFNG-responsive (MCC-336, -350) were transfected with either *NLRC5* or control vectors (empty or GFP). Data visualized in log scale. (**G**) Confirmation of *NLRC5* transfection. Left: Estimated transfection efficiency, as assessed by co-transfection of the pmaxGFP vector with pCMV.2 (second column), FLAG-NLRC5 (third column), or alone (fourth column). Right: qRT-PCR assessment of relative *NLRC5* expression in *NLRC5*-transfected MCC lines compared to wildtype, using two different primer pairs (A and B). Log_2-fold-changes were calculated with the delta-delta Ct method, using *GAPDH* as a housekeeping gene (n =3 biologic replicates: MCC-336, -350, and -367).

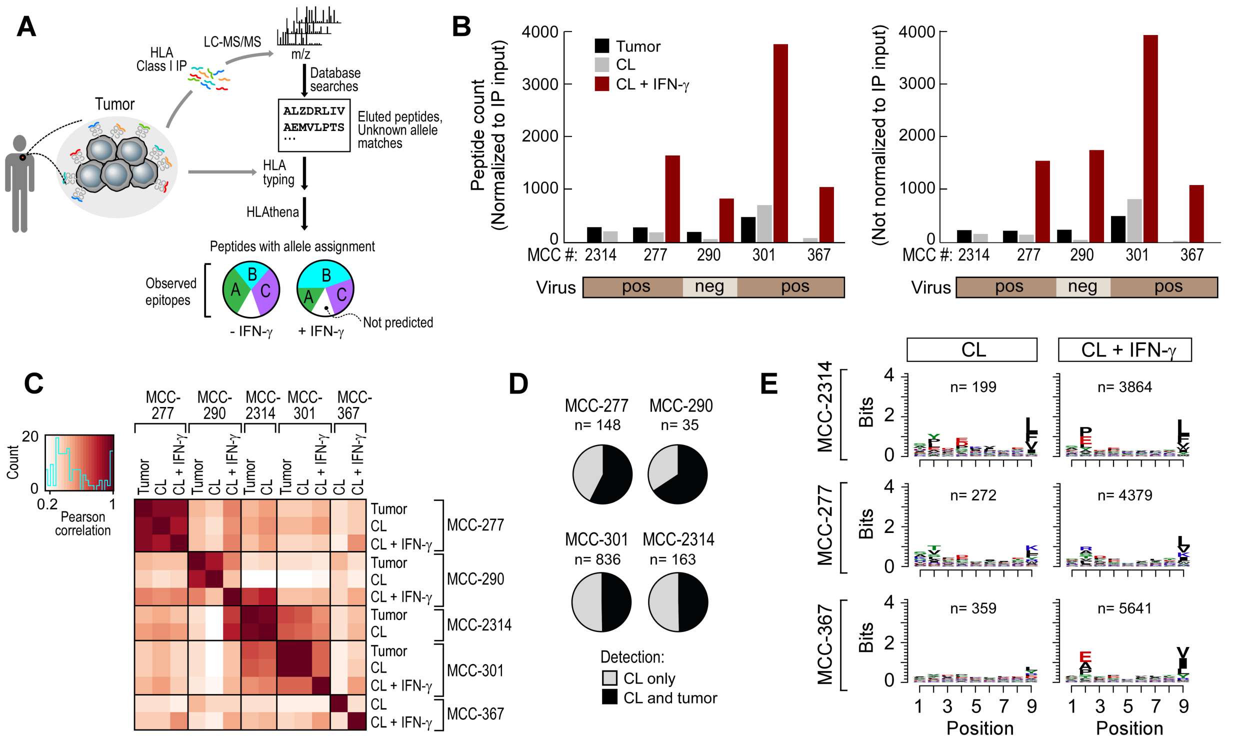

Supplemental Figure 4. Additional immunopeptidome data. (**A**) Schematic representation of immunopeptidome workflow. HLA molecules are immunoprecipitated from tumor and cell line material, peptides are eluted from HLA complex and analyzed by LC-MS/MS. After database searching, peptides are assigned to their most likely allele by prediction in HLAthena. (**B**) Bar charts showing the number of detected peptides in primary tumor, cell line, and IFNG-treated cell lines for select MCC lines. Left: total peptide counts. Right: Peptide counts normalized to IP input. (**C**) Correlation heatmap of peptide sequences in motif space between MCC tumors, cell lines at baseline, and cell lines after IFNG treatment. A subset of sample data in **B** and **C** is also shown in **Figure 3A-B**. (**D**) Pie charts of HLA-I-presented peptides in select MCC cell lines that were also detected in the corresponding tumor sample (black) or were unique to the cell line (gray). (**E**) Motif changes of 9mers between baseline cell line and IFNG-treated cell line samples.

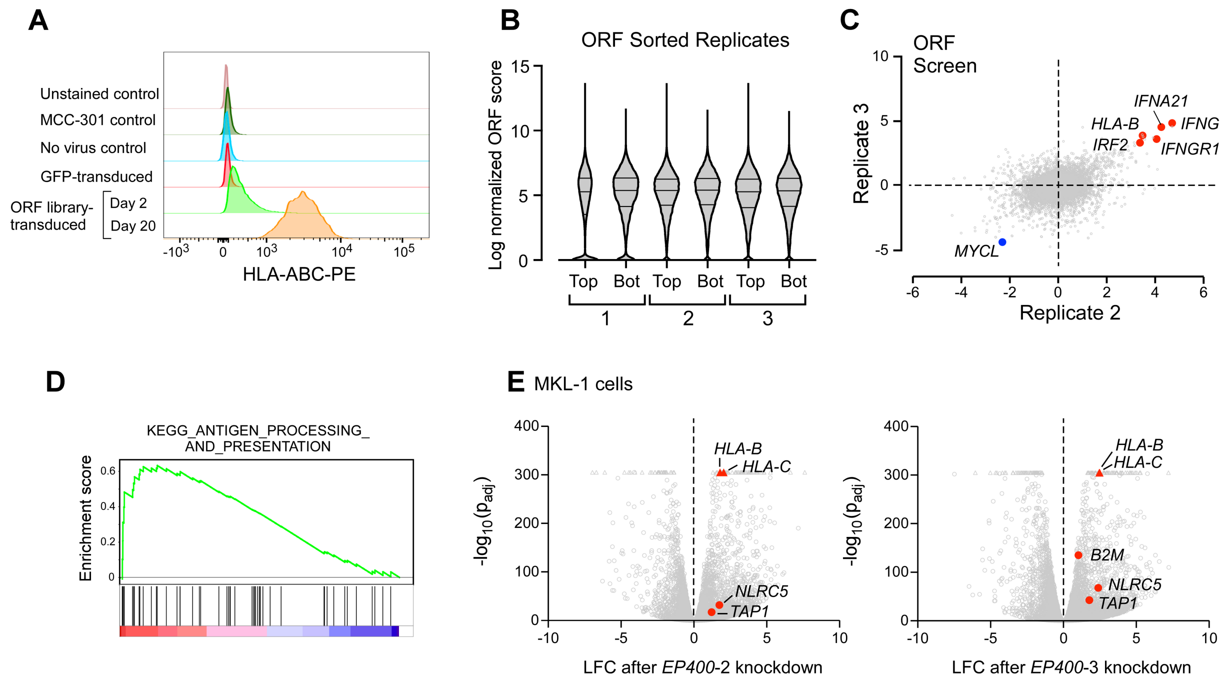

Supplemental Figure 5. ORF screen implicates MYCL as a negative regulator of HLA-I in MCC. (**A**) Flow cytometric assessment of HLA-I surface expression (W6/32 antibody) in MCC-301 cells transduced with the human ORFeome v8.1 library lentivirus. Controls include MCC-301 cells transduced with a GFP ORF virus, a no-virus control, and un-transduced cells. Data visualized with biexponential scaling. (**B**) Violin plot of the $\log_2$ normalized construct abundance scores for each sorted population of the ORF screen. Middle line indicates median; upper and lower lines indicate upper and lower quartiles, respectively. (**C**) Scatterplot of gene-level LFCs (average LFC of all constructs for a given gene) between two replicates of the ORF screen. Notable screen hits are highlighted in red or blue. (**D**) Enrichment of the KEGG term ‘Antigen processing and presentation’ in GSEA analysis of gene upregulated in MKL-1 shMYCL cells relative to a scrambled shRNA control. (**E**) Differential expression analysis of MKL-1 cells transduced with one of two shRNAs against EP400 (shEP400-2 or shEP400-3), compared to a scrambled shRNA control. Red indicates HLA-I genes with LFC > 1 and $p_{adj} < 0.01$. Triangles

indicate genes whose p_{adj} values were reported as zero by DeSeq2, and subsequently plotted at the lowest non-zero p_{adj} value in the dataset.

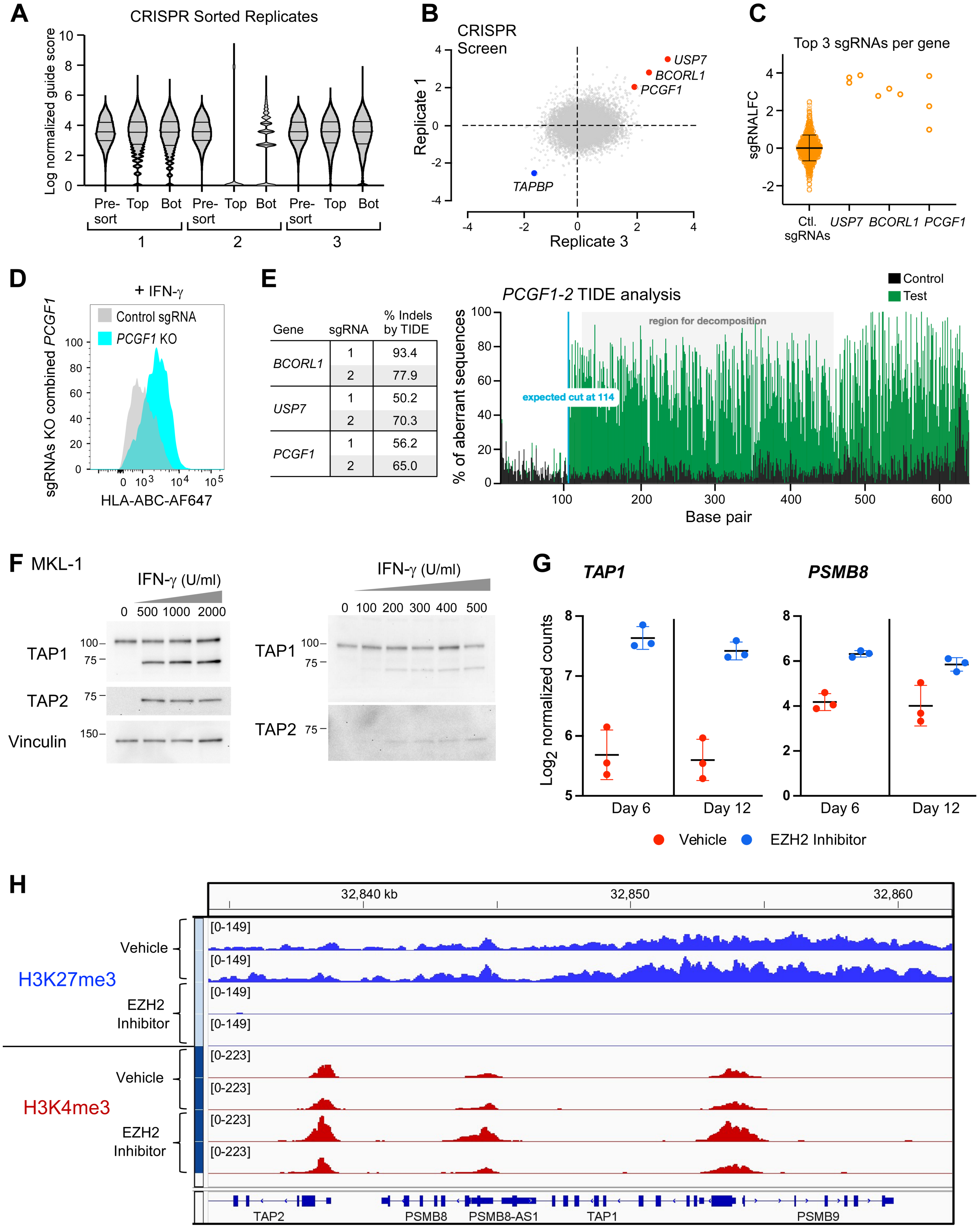
A
CRISPR Sorted Replicates
Log normalized guide score
Pre-sort
Top
Bot
1
2
3
B
CRISPR Screen
USP7
BCORL1
PCGF1
TAPBP
Replicate 1
Replicate 3
C
Top 3 sgRNAs per gene
sgRNALFC
Ctl. sgRNAs
USP7
BCORL1
PCGF1
D
+ IFN-γ
Control sgRNA
PCGF1 KO
sgRNAs KO combined PCGF1
HLA-ABC-AF647
E
Gene
sgRNA
% Indels by TIDE
BCORL1 1 93.4
2 77.9
USP7 1 50.2
2 70.3
PCGF1 1 56.2
2 65.0
PCGF1-2 TIDE analysis
Control
Test
region for decomposition
expected cut at 114
% of aberrant sequences
Base pair
F MKL-1
IFN-γ (U/ml)
0 500 1000 2000
0 100 200 300 400 500
TAP1
TAP2
Vinculin
G
TAP1
PSMB8
Log2 normalized counts
Day 6
Day 12
Vehicle
EZH2 Inhibitor
H
32,840 kb
32,850
32,860
H3K27me3
H3K4me3
Vehicle
EZH2 Inhibitor
[0-149]
[0-223]
TAP2
PSMB8
PSMB8-AS1
TAP1
PSMB9

Supplemental Figure 6. CRISPR screen identifies PRC1.1 as a negative regulator of HLA-I in MCC. (**A**) Violin plot of the log_2 normalized construct abundance scores for each sorted population of the CRISPR screen. Middle line indicates median; upper and lower lines indicate upper and lower quartiles, respectively. (**B**) Scatterplot showing concordance of gene-level LFCs (average LFC of all constructs for a given gene) between two replicates of the CRISPR screen. Notable screen hits are highlighted in red or blue. (**C**) Average LFC enrichment of the 3 highest-scoring sgRNAs for USP7, BCORL1, and PCGF1, with the distribution of a set of control non-targeting or intergenic sgRNAs shown as a reference. (**D**) Flow cytometry for surface HLA-I in a double sgRNA *PCGF1* KO line after IFNG treatment. Data visualized with biexponential scaling. (**E**) TIDE analysis of PRC1.1 single-guide KO lines. Left: the percentage of cells with indels in each knockout line was determined using TIDE software (1). Right: Example TIDE analysis tracing of the *PCGF1* sgRNA #2 KO line in MCC-301. (**F**) Western blot quantification of TAP1 and TAP2 in MKL-1 cells in response to varying concentrations of IFNG. (**G**) RNA-seq normalized expression values of *TAP1* and *PSMB8* in MKL-1 cells treated with 3 μM EZH2 inhibitor (EPZ011989) or vehicle for 6 or 12 days. $P < 0.05$ for both *TAP1* and *PSMB8* at days 6 and 12, as determined by DeSeq2 analysis. (**H**) Genome browser view of H3K37me3 and H3K4me3 histone profiling around the *TAP1/2* and *PSMB8/9* gene loci in MKL-1 cells treated with EZH2 inhibitor (EPZ011989) or vehicle for 6 days.

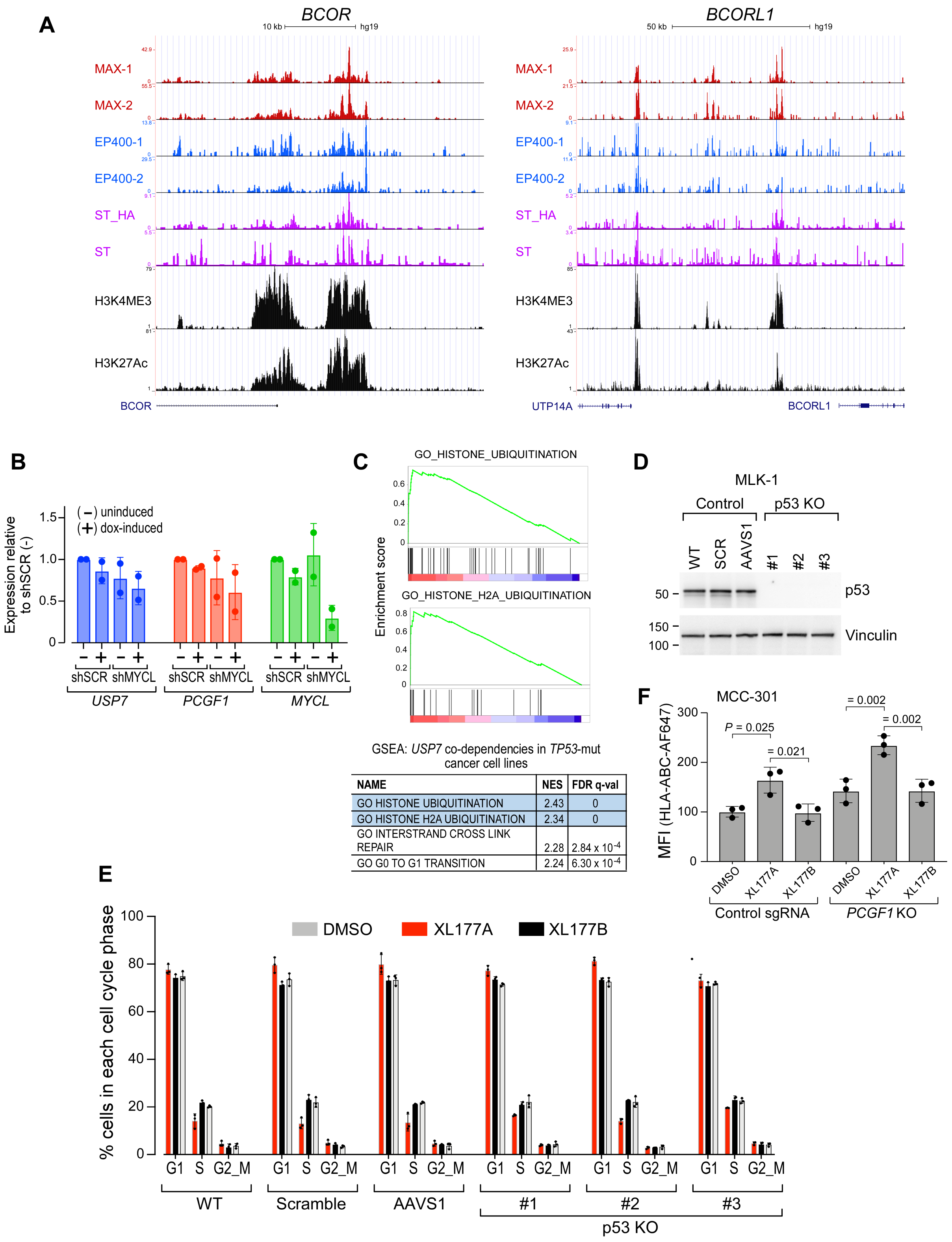
A
BCOR
BCORL1
10 kb
50 kb
hg19
MAX-1
MAX-2
EP400-1
EP400-2
ST_HA
ST
H3K4ME3
H3K27Ac
BCOR
UTP14A
BCORL1
B
(−) uninduced
(+) dox-induced
Expression relative to shSCR (-)
shSCR shMYCL
USP7
PCGF1
MYCL
C
GO_HISTONE_UBIQUITINATION
GO_HISTONE_H2A_UBIQUITINATION
Enrichment score
GSEA: USP7 co-dependencies in TP53-mut cancer cell lines
NAME | NES | FDR q-val
GO HISTONE UBIQUITINATION | 2.43 | 0
GO HISTONE H2A UBIQUITINATION | 2.34 | 0
GO INTERSTRAND CROSS LINK REPAIR | 2.28 | 2.84 x 10-4
GO G0 TO G1 TRANSITION | 2.24 | 6.30 x 10-4
D
MLK-1
Control
p53 KO
WT
SCR
AAVS1
#1
#2
#3
p53
Vinculin
E
% cells in each cell cycle phase
DMSO
XL177A
XL177B
G1 S G2_M
WT
Scramble
AAVS1
#1
#2
#3
p53 KO
F
MCC-301
MFI (HLA-ABC-AF647)
P = 0.025
= 0.021
= 0.002
= 0.002
DMSO
XL177A
XL177B
Control sgRNA
PCGF1 KO

Supplemental Figure 7. Pharmacologic inhibition of USP7 upregulates HLA-I . (**A**) Genome browser view of *BCOR* and *BCORL1* with ChIP-seq tracks for MAX (red), EP-400 (blue), MCPyV ST antigen (pink), and activating histone marks H3K4me3 and H3K27Ac (black). (**B**) qRT-PCR of *USP7* (blue), *PCGF1* (red), and *MYCL* (green) in sh*MYCL* knockdown lines in MKL-1 compared to scrambled shRNA control (n=2 replicates per condition).
(**C**) The GO terms "Histone ubiquitination" and "Histone H2A ubiquitination" are highly enriched within genes that exhibit co-dependency with *USP7* in *TP53*-mut cancer cell lines by GSEA analysis. (**D**) Western blot for p53 in 3 MKL-1 p53 KO lines compared to control lines (WT, SCR, AAVS1). (**E**) Distribution of cell cycle phases, determined by flow cytometry, of MKL-1 p53 KO lines treated with XL177A, XL177B, or DMSO. (**F**) Flow cytometry assessment of surface HLA-I in an MCC-301 *PCGF1* KO line treated with USP7 inhibitor XL177A, control compound XL177B, or DMSO (n=3 per condition). Statistical significance determined by ordinary one-way ANOVA followed by *post hoc* Tukey's multiple comparisons test.

Supplemental Table 1. MCC cell line panel sequencing.
WES mutation calls, copy number variations, and RNA-seq differential expression analysis +/- IFNG for the panel of newly generated MCC cell lines.

Supplemental Table 2. CRISPR and ORF screen gene rankings.
Gene-level rankings of the CRISPR and ORF screens, GSEA analysis of screen hits, and list of genes detected in the MCC HLA CRISPR screen that have been found in other HLA CRISPR screens.

Supplemental Table 3. MKL-1 shRNA GSEA
Gene set enrichment analysis of genes more highly expressed in the MKL-1 shMYCL cell line compared to a scrambled shRNA control line.

Supplemental Table 4. USP7 co-dependency rankings and GSEA analysis.
List of genes that exhibited co-dependency with USP7 within the Cancer Dependency Map.

Supplemental Table 5. HLA-I immunopeptidomes of MCC-301 treated with USP7 inhibitors
List of HLA-I-presented peptides with significantly different abundances between MCC-301 cells treated with XL177A, XL177B, or untreated cells.

Supplemental Table 6. HLA typing.
HLA typing for 7 of the 11 MCC lines for which whole-exome sequencing data was available.

Supplemental Table 7. Oligos and primers.

Supplemental Methods

Media formulations

Other media formulations tested during cell culture optimization included StemFlex (Gibco); Neurobasal (Gibco) with 0.02% heparin (StemCell Technologies), 20 ng/mL hEGF (Miltenyi Biotec), and 20 ng/mL hFGF2 (Miltenyi Biotec); DMEM GlutaMAX (Gibco) supplemented with 10% FBS (Gibco), 1% penicillin/streptomycin (Gibco), 1mM sodium pyruvate (Life Technologies), 10mM HEPES (Life Technologies), and 55nM β-mercaptoethanol (Gibco); and RPMI-1640 (Gibco) with 20% FBS (Gibco) and 1% penicillin/streptomycin (Gibco).

Histology and Immunohistochemistry

All IHC was performed on the Leica Bond III automated staining platform. From the cell lines, up to 10 million MCC cells were pelleted, fixed in formaldehyde, washed with PBS, and mounted on a paraffin block. For single stains, 5-micron sections were cut and stained for SOX2 or CK20. The Leica Biosystems Refine Detection Kit was used with citrate antigen retrieval for SOX2 (Abcam #97959, polyclonal, 1:100 dilution) and with EDTA antigen retrieval for Cytokeratin 20 (CK20; Dako #M7019, clone Ks20.8, 1:50 dilution). For dual immunohistochemical staining of the archival tumor specimens, we used MCC marker SOX2 (CST, D6D9, 1:50 dilution; red chromogen) and either HLA class I (Abcam, EMR8-5, 1:6,000 dilution; brown chromogen) or HLA class II (Dako M0775, CR3/43, 1:750 dilution; brown chromogen) using an automated staining system (Bond III, Leica Biosystems) according to the manufacturer's protocol, as previously described (2). The proportion of SOX2+ MCC cells that

exhibited HLA I or HLA II membranous staining was evaluated by consensus of two board-certified pathologists.

Immunofluorescence

Staining was performed overnight on BOND RX fully automated stainers (Leica Biosystems). 5-μm thick formalin-fixed paraffin-embedded tumor tissue sections were baked for 3 hours at 60°C before loading into the BOND RX. Slides were deparaffinized (BOND DeWax Solution, Leica Biosystems, Cat. AR9590) and rehydrated through a series of graded ethanol to deionized water. Antigen retrieval was performed in BOND Epitope Retrieval Solution 1 (ER1; pH 6) or 2 (ER2; pH 9) (Leica Biosystems, Cat. AR9961, AR9640) at 95°C. Deparaffinization, rehydration and antigen retrieval were all pre-programmed and executed by the BOND RX. Next, slides were serially stained with primary antibodies for: SOX2 (clone B6D9, Cell Signaling, dilution 1:200; Opal 690 1:100), CD8 (clone 4B11, Leica, dilution 1:200; Opal 480 1:150), PD-L1 (clone E1L3N, Cell Signaling, dilution 1:300; Opal 520 1:150), and PD-1 (clone EPR4877[2], Abcam, dilution 1:300; Opal 620 1:300) with ER1 for 20 min; and FOXP3 (clone D608R, Cell Signaling, dilution 1:100; Opal 570 1:300) with ER2 solution for 40 min. Each primary antibody was incubated for 30 minutes. Subsequently, anti-mouse plus anti-rabbit Opal Polymer Horseradish Peroxidase (Akoya Biosciences, Cat. ARH1001EA) was applied as a secondary label with an incubation time of 10 minutes. Signal for antibody complexes was labeled and visualized by their corresponding Opal Fluorophore Reagents (Akoya) by incubating the slides for 10 minutes. Slides were incubated in Spectral DAPI solution (Akoya) for 10 minutes, air dried, and mounted with Prolong Diamond Anti-fade mounting medium (Life Technologies, Cat. P36965) and imaged using the Vectra Polaris multispectral imaging platform (Vectra Polaris, Akoya

Biosciences). Representative tumor regions of interest were identified by the pathologist and 2-6 fields of view were acquired per sample. Images were spectrally unmixed and cell identification was performed using the supervised machine learning algorithms within Inform 2.4 (Akoya) with pathologist supervision as previously described (2).

Whole exome sequencing analysis

BAM files were downloaded from the Broad Genomics Firecloud/Terra platform. GATK version 4.1.2.0(3) was used to call mutations from reference on normal BAMs with Mutect2 command (4) using a max MNP distance of 0, build a panel of normals from VCF files of called normal mutations using the CreateSomaticPanelOfNormals command, and call mutations between pairs of both tumor and cell line with compared to their respective normal counterpart using the Mutect2 command. For these steps, the following annotations were used: b37 reference sequence downloaded from ftp://ftp.broadinstitute.org/bundle/b37/human_g1k_v37.fasta, germline resource VCF downloaded from ftp://ftp.broadinstitute.org/bundle/beta/Mutect2/af-only-gnomad.raw.sites.b37.vcf.gz, and intervals list downloaded from https://github.com/broadinstitute/gatk/blob/master/src/test/resources/large/whole_exome_illumina_coding_v1.Homo_sapiens_assembly19.targets.interval_list. Called variants were filtered with the GATK FilterMutectCalls command, and variants labeled as PASS were extracted and included in downstream analyses. Next, VCF files of passing variants were annotated as MAF files using vcf2maf version 1.16.17 and Variant Effect Predictor version 95 (5). R Bioconductor package maftools (6) was used to generate oncoplots of mutations by gene and sample.

RNA-seq analysis of MCC cell lines

FASTQ files from fibroblasts, keratinocytes, MKL-1, and WaGa were aligned using STAR version 2.7.3a (7), using the index genome reference file downloaded from ftp://ftp.ebi.ac.uk/pub/databases/gencode/Gencode_human/release_19/GRCh37.p13.genome.fa.gz, the transcript annotation file downloaded from https://data.broadinstitute.org/snowman/hg19/star/gencode.v19.annotation.gtf, and with the following options: --twopassMode Basic, --outSAMstrandField intronMotif, --alignIntronMax 1000000, --alignMatesGapMax 1000000, --sjdbScore 2, --outSAMtype BAM Unsorted, --outSAMattributes NH HI NM MD AS XS, --outFilterType BySJout, --outSAMunmapped Within, --genomeLoad NoSharedMemory, --outFilterScoreMinOverLread 0, --outFilterMatchNminOverLread 0, --outFilterMismatchNmax 999, and outFilterMultimapNmax 20. Duplicates were marked with picard MarkDuplicates version 2.22.0-SNAPSHOT. RNA-sequencing BAM files for MCC tumor and cell line samples were downloaded from the Broad Genomics Firecloud/Terra platform

Gene counts were obtained from BAM files using featureCounts version 2.0.0 (8). Very lowly expressed genes with average count across samples less than 1 were excluded from analysis. Between-sample distance metrics (**Figure 1C** from main text) were computed using the Euclidean distance on the vectors of variance-stabilized counts obtained from the vst function in the DESeq2 R Bioconductor package (7, 9).

Differential expression analysis was carried out between IFNG plus and minus samples (adjusting for viral status as a covariate) using the negative binomial GLM Wald test of DESeq2, where significance was assessed using the p-values adjusted for multiple comparisons under default settings. To account for potential global gene expression differences among sample groups, RUVg (10) was used to estimate latent factors of unwanted variation from the list of

housekeeping genes downloaded from https://www.tau.ac.il/~elieis/HKG/HK_genes.txt. The largest factor of unwanted variation was then used as a covariate in the DESeq2 models to adjust for latent variation unrelated to library size. The normalized counts adjusted for the latent factors of variation returned by RUVg were visualized in **Figure 2A** (main text).

MCPyV viral DNA and RNA detection

DNA detection of MCPyV in MCC tumor samples was performed with ViroPanel as previously described (11). For viral transcript quantification of RNA-seq, the Merkel Cell Polyomavirus reference sequence was downloaded from https://www.ebi.ac.uk/ena/data/view/EU375804&display=fasta. Reads that did not map to the human reference sequence were extracted from RNA-seq and ViroPanel BAM files of tumor and cell line using SAMtools view version 1.10 (12) and realigned to a modified Merkel Cell Polyomavirus reference sequence (HM355825.1, recircularized such that the reference sequence ends when the VP2 coding sequence ends) using BWA version 0.7.17-r1188 (13). Coverage at each position was assessed with samtools using the command `samtools depth -aa -d0`, and coverage depth was plotting in R version 3.5.1 using the ggplot2 and gggenes packages.

Single-cell RNA sequencing

MCC-336 and MCC-350 tumor samples were thawed and washed twice in RPMI and 10% FBS before undergoing dead cell depletion (Miltenyi 130-090-101). Viable MCC cells were resuspended in PBS with 0.04% BSA at 1,000 cells/µL. 17,000 cells were loaded onto a 10x Genomics Chromium^{TM} instrument (10x Genomics) according to the manufacturer's instructions. The scRNAseq libraries were processed using Chromium^{TM} single cell 5' library &

gel bead kit (10x Genomics). Quality control for amplified cDNA libraries and final sequencing libraries were performed using Bioanalyzer High Sensitivity DNA Kit (Agilent). ScRNAseq libraries were normalized to 4nM, pooled, and sequenced on Illumina NovaSeq S4 platform (150 bp paired reads and 8 bp index read). Reads were demultiplexed and aligned to hg19 using Cell Ranger (v. 3.0.2) (14) and the transcript quantities were co-analyzed using the Seurat (v. 3.1.5) R package (15). Only cells expressing >1,500 and <7,500 genes and <10 % mitochondrial genes were kept for further analysis, leaving a total of 15,808 cells sequenced to a mean depth of 4,231.9 genes/cell. The data was normalized and the top 2,000 variable features were identified. Subsequently, the data was scaled while regressing out variation from gene count, mitochondrial percentage, and cell cycle stage. This was followed by principal component analysis, batch correction using Harmony (v. 1.0) (16), UMAP analysis, and finally, Louvain clustering at resolution = 0.3. The immune cell cluster was identified by the expression of *CD45* (*PTPRC*) and MCC clusters were identified by expression of *ATOH1*, *SYP,* and *SOX2*.

Whole genome sequencing and copy number analysis

Whole genome sequencing was performed by Admera Health. Paired-end sequencing was performed on an Illumina HiSeq X Ten with a read length of 150bp. Reads were quality and adapter trimmed using TrimGalore with default settings. Trimmed reads were aligned against a fusion reference containing hg38 and MCPyV (NCBI accession number: NC_010277) using bowtie2 –very-sensitive. Copy number variant analysis was performed with GATK4 CNV recommended practices. A panel of normals was generated from 17 normal blood whole genomes to call CNVs from tumors. All CNV calls that mapped to hg38 were visualized using

the Integrative Genomics Viewer from Broad Institute (http://software.broadinstitute.org/software/igv/).

NLRC5 transfection experiments

MCC cell lines 336, 350, and 367 were resuspended in 100uL Nucleofection Solution V (Cat no: VCA-1003). 1 μg of FLAG-NLRC5 vector (Addgene #37521) and the control backbone vector pCMV.2 were co-transfected with 0.5 μg of pmaxGFP vector, using the Lonza Nucleofector 2b device (cat no: AAB-1001), program X-001. Transfection efficiency was estimated by GFP expression. To confirm *NLRC5* overexpression, qRT-PCR was performed on RNA from wildtype and *NLRC5*-transfected MCC lines, using SYBR Green to quantiy NLRC5 and GAPDH (primers listed in **Supplemental Table 7**). Relative expression was calculated using the delta-delta Ct method. HLA-I flow cytometry was performed as described elsewhere in the **Methods**.

Immunoprecipitation, mass spectrometry analysis, and peptide identification

Up to 40 million or 0.2g of MCC cells were immunoprecipitated. Briefly, MCC cells were harvested and lysed in ice-cold lysis buffer containing 40M Tris (pH 8.0), 1mM EDTA (pH 8.0), 0.1M sodium chloride, Triton X-100, 0.06M octyl β-d-glucopyranoside, 100 U/mL DNAse I, 1mM phenylmethanesulfonyl fluoride (all from Sigma Aldrich), and protease inhibitor cocktail (Roche Diagnostics). Cell lysate was centrifuged at 12,700 rpm at 4°C for 22 min. Lysate supernatant was coupled with Gammabind Plus sepharose beads (GE Healthcare) and incubated with 10 μg of HLA-I antibody (Clone W6/32, Santa Cruz Biotechnologies) at 4°C under rotary agitation for 3h. After incubation, the lysate-bead-antibody mixture was briefly centrifuged and the supernatant was discarded. Beads were washed with lysis buffer, consisting of wash buffer containing 40mM Tris (pH 8.0), 1mM EDTA (pH 8.0), 0.1M sodium chloride, 0.06M octyl β-d-glucopyranoside,

and 20mM Tris buffer, without protease inhibitors. Gel loading tips (Fisherbrand) were used to remove as much fluid from beads as possible. Peptides of up to three immunoprecipitations were combined, acid eluted, and analyzed using LC/MS-MS as described previously (17, 18). Briefly, peptides were resuspended in 3% acetonitrile with 5% formic acid and loaded onto an analytical column (20-30 cm with 1.9 µm C18 Reprosil beads, Dr. Maisch HPLC GmbH, packed in-house). Peptides were eluted in a 6-30% gradient (EasyLC 1000 or 1200, Thermo Fisher Scientific) and analyzed on a QExactive Plus, Fusion Lumos, or Orbitrap Exploris 480 (Thermo Fisher Scientific). For Lumos measurements, peptides were also subjected to fragmentation if they were singly charged. For Orbitrap Exploris measurements (2 immunoprecipitations pooled, +/- IFNG, **Figure 3**) and detection of the large T antigen peptide (3 immunoprecipitations of the MCC-367 cell line treated with IFNG) peptides were further fractionated using stage tip basic reverse phase separation with 2 punches of SDB-XC material (Empore 3M) and increasing concentrations of acetonitrile (5%, 10% and 30% in 0.1% NH4OH, pH 10). Fractions were analyzed on a Fusion Lumos or Orbitrap Exploris 480 equipped with a FAIMSpro interface (19).

Immunopeptidomes of USP7 inhibitor treated cell lines were eluted as described above, followed by labeling with TMT6 reagent (Thermo Fisher; 126-USP7iA, 127-WT, 128 USP7iA, 129 WT, 130-USP7iB, 131 USP7iB) and then pooled for subsequent fractionation using basic reversed phase fractionation with increasing concentrations of acetonitrile (10%, 15% and 50%) in 5 mM ammonium formate (pH 10) and analysis on an Orbitrap Exploris 480 with FAIMSpro. Data acquisition parameters were as above with NCE set to 34 and 2 second dynamic exclusion.

Mass spectra were interpreted using Spectrum Mill software package v7.1 pre-Release (Broad Institute, Cambridge, MA). MS/MS spectra were excluded from searching if they did not have a precursor MH+ in the range of 600-4000, had a precursor charge >5, or had a minimum of <5

detected peaks. Merging of similar spectra with the same precursor m/z acquired in the same chromatographic peak was disabled. MS/MS spectra were searched against a protein sequence database that contained 90,904 entries, including all UCSC Genome Browser genes with hg19 annotation of the genome and its protein coding transcripts (52,788 entries), common human virus sequences (30,181 entries), recurrently mutated proteins observed in tumors from 26 tissues (4,595 entries), 264 common laboratory contaminants as well as protein sequences containing somatic mutations detected in MCC cell lines (3,076 entries). MS/MS search parameters included: no-enzyme specificity; ESI-QEXACTIVE-HCD-HLA-v3 instrument scoring; fixed modification: cysteinylation of cysteine; variable modifications: oxidation of methionine, carbamidomethylation of cysteine and pyroglutamic acid at peptide N-terminal glutamine; precursor mass tolerance of ±10 ppm; product mass tolerance of ±10 ppm, and a minimum matched peak intensity of 30%. Peptide spectrum matches (PSMs) for individual spectra were automatically designated as confidently assigned using the Spectrum Mill auto-validation module to apply target-decoy based FDR estimation at the PSM level of <1% FDR. Peptide auto-validation was done separately for each sample with an auto thresholds strategy to optimize score and delta Rank1 – Rank2 score thresholds separately for each precursor charge state (1 through 4) across all LC-MS/MS runs per sample. Score threshold determination also required that peptides had a minimum sequence length of 7, and PSMs had a minimum backbone cleavage score of 5. Peptide and PSM exports were filtered for contaminants including potential carry over tryptic peptides and peptides identified in a blank bead sample. For TMT-labeled samples, peptides derived from keratin proteins were removed and TMT intensity values were normalized to the global median. P-values were calculated using in house software based on the *limma* package in R.

Whole proteome analysis and interpretation

Protein expression of MCC cell lines was assessed as described previously (20). Briefly, cell pellets of MCC cell lines with and without IFNG treatment were lysed in 8M Urea and digested to peptides using LysC and Trypsin (Promega). 400 µg peptides were labeled with TMT10 reagents (Thermo Fisher, 126-MCC-290, 127N – MCC-350 _IFN, 127C MCC-275_IFN, 128N MCC-275, 128C MCC-350, 129N_MCC-301_IFN, 129C – MCC-277_IFN, 130N-MCC-290_IFNy, 130C MCC-277, 131 MCC-301) and then pooled for subsequent fractionation and analysis. Pooled peptides were separated into 24 fractions using offline high pH reversed phase fractionation. 1 µg per fraction was loaded onto an analytical column (20-30 cm with 1.9 µm C18 Reprosil beads [Dr. Maisch HPLC GmbH], packed in-house, PicoFrit 75 µM inner diameter, 10 µM emitter [New Objective]). Peptides were eluted with a linear gradient (EasyNanoLC 1000 or 1200, Thermo Scientific) ranging from 6-30% Buffer B (either 0.1% formic acid or 0.5% AcOH and 80% or 90% acetonitrile) over 84 min 30-90% Buffer B over 9 min, and held at 90% Buffer B for 5 min at 200 nl/min. During data dependent acquisition, peptides were analyzed on a Fusion Lumos (Thermo Scientific). Full scan MS was acquired at a 60,000 from 300 - 1,800 m/z. AGC target was set to 4e5 and 50 ms. The top 20 precursors per cycle were subjected to HCD fragmentation at 60,000 resolution with an isolation width of 0.7 m/z, 34 NCE, 3e4 AGC target, and 50ms max injection time. Dynamic exclusion was enabled with a duration of 45 sec.

Spectra were searched using Spectrum Mill against the database described above excluding MCC variants, specifying Trypsin/allow P (allows K-P and R-P cleavage) as digestion enzyme and allowing 4 missed cleavages, and ESI-QEXACTIVE-HCD-v3. Carbamidomethylation of cysteine was set as a fixed modification. TMT labeling was required at lysine, but peptide N-

termini were allowed to be either labeled or unlabeled. Variable modifications searched include acetylation at the protein N-terminus, oxidized methionine, pyroglutamic acid, deamidated asparagine, and pyrocarbamidomethyl cysteine. Match tolerances were set to 20 ppm on MS1 and MS2 level. PSMs score thresholding used the Spectrum Mill auto-validation module to apply target-decoy based FDR in 2 steps: at the peptide spectrum match (PSM) level and the protein level. In step 1 PSM-level auto-validation was done first using an auto-thresholds strategy with a minimum sequence length of 8; automatic variable range precursor mass filtering; and score and delta Rank1 – Rank2 score thresholds optimized to yield a PSM-level FDR estimate for precursor charges 2 through 4 of <1.0% for each precursor charge state in each LC-MS/MS run. To achieve reasonable statistics for precursor charges 5-6, thresholds were optimized to yield a PSM-level FDR estimate of <0.5% across all LC runs per experiment (instead of per each run), since many fewer spectra are generated for the higher charge states. In step 2, protein-polishing auto-validation was applied to each experiment to further filter the PSMs using a target protein-level FDR threshold of zero, the protein grouping method expand subgroups, top uses shared (SGT) with an absolute minimum protein score of 9. TMT10 reporter ion intensities were corrected for isotopic impurities in the Spectrum Mill protein/peptide summary module using the afRICA correction method which implements determinant calculations according to Cramer's Rule (21) and correction factors obtained from the reagent manufacturer's certificate of analysis (https://www.thermofisher.com/order/catalog/product/90406) for lot number TB266293.

Screen Data Analysis

FASTQ reads were converted to log_2-normalized scores for each construct using PoolQ v2.2.0 (https://portals.broadinstitute.org/gpp/public/software/poolq). Log_2-fold changes (LFCs) between

the normalized count scores of the HLA-I-high and HLA-I-low populations were calculated for each construct.

For the ORF screen, ORF constructs were then ranked based on their median LFC values (https://portals.broadinstitute.org/gpp/public/analysis-tools/crispr-gene-scoring). Sample quality for each sorted population was assessed by calculating log-normalized ORF construct scores ($\log_2$ (ORF construct reads / total reads $\times$ 10^6 + 1) and confirming than the mean construct frequency was no less than 10% of the expected frequency if all constructs were equally represented (corresponding to mean log-normalized score cutoff of 2.84) (**Supplemental Figure 5B**).

For the CRISPR screen, using equivalent cutoff criteria as above, replicate 2 was discarded because the mean log-normalized score of the replicate 2 HLA-I-high sorted population was only 0.413 (**Supplemental Figure 6A**). Subsequently, LFC values for each sgRNA were averaged between replicate 1 and 3 only and then input into the STARS software (22).

MKL-1 shMYCL and WaGa shST/LT cell line generation and RNA-seq

A scramble shRNA constitutively expressed from the lentiviral PLKO vector (shScr) has been reported before (Addgene #1864). The MYCL and EP400 shRNA target sequences were designed using Block-iT RNAi Designer (Life Technologies). MYCL target – GACCAAGAGGAAGAATCACAA; shEP400-2 target – GCTGCGAAGAAGCTCGTTAGA, shEP400-3 target – GGAGCAGCTTACACCAATTGA. Annealed forward and reverse oligos of shScr, shMYCL, shEP400-2, and shEP400-3 (**Supplemental Table 7**) were cloned between AgeI/EcoRI sites of the doxycycline inducible shRNA vector Tet-pLKO-puro (a gift from Dmitri

Wiederschain, Addgene #21915). 293T cells were transfected with the Tet-PLKO-puro plasmids plus psPAX2 packaging and VSV-G envelope plasmids (Addgene #12260 and #12259) to generate lentiviral particles for MKL-1 cell transduction. Transduced MKL-1 cells were selected with 1 µg puromycin for 4 days to generate Dox-inducible MKL-1 shScr, shMYCL, shEP400-2, and shEP400-3 lines. The Dox-inducible WaGa shST/LT line was a gift from Roland Houben (23).

For RNA-seq, cells were treated with dox as follows: MKL-1 shMYCL and shScr – 2 days Dox, MKL-1 shEP400-2, -3 and shScr - 6 days Dox, WaGa shST/LT cells with or without Dox - 6 days. Total RNA was extracted using RNeasy Plus Mini Kit (Qiagen). mRNA was isolated with NEB- Next Poly(A) mRNA Magnetic Isolation Module (New England BioLabs). Sequencing libraries were prepared with NEBNext mRNA library Prep Master Mix Set for Illumina (New England BioLabs) and passed Qubit, Bioanalyzer, and qPCR QC analyses. 50 cycles single-end sequencing was performed on the Illumina HiSeq 2000 system. Reads were mapped to the hg19 genome by TOPHAT. HTSeq was used to create a count file containing gene names (24). The R package DESeq2 was used to normalize counts and calculate total reads per million (TPM) and determine differential gene expression. Quality control was performed by inspecting a MA plot of differentially expressed genes. RNA-seq data are available from the Gene Expression Omnibus with accession number GSE69878.

MCC Tumor RNA-seq Cohort

Tumor biopsies were collected from 52 patients at Dana-Farber Cancer Institute. RNA-seq libraries were prepared with the NEBNext Ultra II RNA Library Prep Kit for Illumina (NEB). Paired-end sequencing (150 cycles) was performed on the NovaSeq (Novogene). Sequencing

data were broadly assessed for quality via FastQC (https://www.bioinformatics.babraham.ac.uk/projects/fastqc/). Samples passing quality control were quantified to the transcript level via Salmon (25) utilizing Ensembl gene annotations for the GRCh38.p13 genome assembly. Normalized gene-level counts were prepared with TxImport and DESeq2 (9, 26). To identify MCPyV+ versus MCPyV- samples, paired-end reads were mapped to the MCPyV genome (R17b isolate) via BWA (13) and those sample containing MCPyV-specific reads (>100) were considered MCPyV+. Z-scores of the $\log_2$-normalized gene-level counts were calculated. One tumor sample was discarded as an outlier because the z-score was >3.5 or < -3.5 in 7 of the 18 class I genes analyzed. The remaining 51 tumor samples were clustered by Euclidian distance to generate the RNA-seq heatmap. Tumor purity was determined using the ESTIMATE R Package (27). Tumor purity percentage was calculated from the ESTIMATE score using the equation: $\cos(0.6049872018+0.0001467884 \times \text{ESTIMATE score})$ as published.

EZH2 Inhibitor Experiments

MKL-1 (MCPyV+) cells were treated for 6 or 12 days with either 3 μM EZH2 inhibitor (EPZ011989) or vehicle (Vhc), after which RNA-seq was performed as previously described (28). By DeSeq2 analysis, *TAP1* and *PSMB8* were the only two class I genes that exhibited significant p-value < 0.05 (p_adj), $\log_2$-fold-change > 1, and baseMean > 40 at both day 6 and 12. Histone profiling using CUT&RUN was performed after 6 days of treatment with EPZ011989 or vehicle, as previously described (28).

Dependency Map Correlations

The DepMap 20Q2 CRISPR dependency data were downloaded from www.depmap.org/portal/download. *TP53* mutation status was assigned using the Cell-Line Selector tool on the DepMap Portal based on criteria of at least one coding mutation. Pearson coefficients were calculated using *test.cor* in R, and two-sided p-values outputted by this function were converted into FDR using *p.adjust*. Plots were generated using *ggplot2, tidyverse, gridExtra, cowplot*, and *scales*. GSEA was performed using a gene list ranked by -log(p-val) multiplied by (-1) if the Pearson correlation was negative.

Cell cycle analysis

1 million MKL-1 control or p53 KO cells were plated and treated with DMSO, XL177A (100nM) or XL177B (100nM) for three days. During the last hour of the three- day treatment, the cells were pulsed with 10μM EdU nucleotide. The cells were collected by centrifugation, treated with Accutase™ (Stem Cell Technologies) to break apart clumps, washed with PBS and fixed using 4% Formaldehyde solution in PBS at Room temperature for 15 mins. Cells were washed with 1% BSA in PBS and resuspended in 70% ice cold ethanol and incubated at -20°C overnight for additional fixing and permeabilization. The cells were stored in 70% ethanol at -20°C until the day the data was acquired. On the day of data acquisition, the cells were collected by centrifugation and washed twice with PBS. The incorporated EdU in the cells were labeled with a CLICK reaction cocktail (1 mM $CuSO_4$, 100 μM THPTA, 100 mM sodium ascorbate, and 2.2 μM Alexa 647 azide in PBS) at room temperature with rocking for 30 minutes. The samples were then washed with 1% BSA in PBS once followed by two washes with PBS and incubated with a 1 μg/ml DAPI, 100 ng/ml RNase A solution for one hour at Room temperature to stain the DNA. The samples were then passed through strainer tubes and analyzed using a BD Fortessa analyzer. The flow cytometry data was

analyzed using the FlowJo Software. The percentage of cells in each cell cycle phase was represented using GraphPad PRISM software.

References

1. Brinkman EK, Chen T, Amendola M, and van Steensel B. Easy quantitative assessment of genome editing by sequence trace decomposition. *Nucleic acids research.* 2014;42(22):e168.
2. Hu Z, Leet DE, Allesøe RL, Oliveira G, Li S, Luoma AM, et al. Personal neoantigen vaccines induce persistent memory T cell responses and epitope spreading in patients with melanoma. *Nature medicine.* 2021;27(3):515-25.
3. do Valle ÍF, Giampieri E, Simonetti G, Padella A, Manfrini M, Ferrari A, et al. Optimized pipeline of MuTect and GATK tools to improve the detection of somatic single nucleotide polymorphisms in whole-exome sequencing data. *BMC Bioinformatics.* 2016;17(12):341.
4. Benjamin D, Sato T, Cibulskis K, Getz G, Stewart C, and Lichtenstein L. Calling Somatic SNVs and Indels with Mutect2. *bioRxiv.* 2019:861054.
5. McLaren W, Gil L, Hunt SE, Riat HS, Ritchie GRS, Thormann A, et al. The Ensembl Variant Effect Predictor. *Genome Biology.* 2016;17(1):122.
6. Mayakonda A, Lin DC, Assenov Y, Plass C, and Koeffler HP. Maftools: efficient and comprehensive analysis of somatic variants in cancer. *Genome research.* 2018;28(11):1747-56.
7. Dobin A, Davis CA, Schlesinger F, Drenkow J, Zaleski C, Jha S, et al. STAR: ultrafast universal RNA-seq aligner. *Bioinformatics (Oxford, England).* 2013;29(1):15-21.
8. Liao Y, Smyth GK, and Shi W. featureCounts: an efficient general purpose program for assigning sequence reads to genomic features. *Bioinformatics (Oxford, England).* 2014;30(7):923-30.
9. Love MI, Huber W, and Anders S. Moderated estimation of fold change and dispersion for RNA-seq data with DESeq2. *Genome Biology.* 2014;15(12):550.
10. Risso D, Ngai J, Speed TP, and Dudoit S. Normalization of RNA-seq data using factor analysis of control genes or samples. *Nature biotechnology.* 2014;32(9):896-902.
11. Starrett GJ, Thakuria M, Chen T, Marcelus C, Cheng J, Nomburg J, et al. Clinical and molecular characterization of virus-positive and virus-negative Merkel cell carcinoma. *Genome Medicine.* 2020;12(1):30.
12. Li H, Handsaker B, Wysoker A, Fennell T, Ruan J, Homer N, et al. The Sequence Alignment/Map format and SAMtools. *Bioinformatics (Oxford, England).* 2009;25(16):2078-9.
13. Li H, and Durbin R. Fast and accurate short read alignment with Burrows-Wheeler transform. *Bioinformatics (Oxford, England).* 2009;25(14):1754-60.
14. Zheng GXY, Terry JM, Belgrader P, Ryvkin P, Bent ZW, Wilson R, et al. Massively parallel digital transcriptional profiling of single cells. *Nature Communications.* 2017;8(1):14049.
15. Stuart T, Butler A, Hoffman P, Hafemeister C, Papalexi E, Mauck WM, 3rd, et al. Comprehensive Integration of Single-Cell Data. *Cell.* 2019;177(7):1888-902.e21.
16. Korsunsky I, Millard N, Fan J, Slowikowski K, Zhang F, Wei K, et al. Fast, sensitive and accurate integration of single-cell data with Harmony. *Nature methods.* 2019;16(12):1289-96.
17. Abelin JG, Keskin DB, Sarkizova S, Hartigan CR, Zhang W, Sidney J, et al. Mass Spectrometry Profiling of HLA-Associated Peptidomes in Mono-allelic Cells Enables More Accurate Epitope Prediction. *Immunity.* 2017;46(2):315-26.
18. Sarkizova S, Klaeger S, Le PM, Li LW, Oliveira G, Keshishian H, et al. A large peptidome dataset improves HLA class I epitope prediction across most of the human population. *Nature biotechnology.* 2020;38(2):199-209.
19. Klaeger S, Apffel A, Clauser KR, Sarkizova S, Oliveira G, Rachimi S, et al. Optimized liquid and gas phase fractionations increase HLA-peptidome coverage for primary cell and tissue samples. *Molecular Cellular Proteomics.* Under Revision.
20. Mertins P, Tang LC, Krug K, Clark DJ, Gritsenko MA, Chen L, et al. Reproducible workflow for multiplexed deep-scale proteome and phosphoproteome analysis of tumor tissues by liquid chromatography-mass spectrometry. *Nature protocols.* 2018;13(7):1632-61.
21. Shadforth IP, Dunkley TPJ, Lilley KS, and Bessant C. i-Tracker: For quantitative proteomics using iTRAQ™. *BMC Genomics.* 2005;6(1):145.
22. Doench JG, Fusi N, Sullender M, Hegde M, Vaimberg EW, Donovan KF, et al. Optimized sgRNA design to maximize activity and minimize off-target effects of CRISPR-Cas9. *Nature biotechnology.* 2016;34(2):184-91.

23. Hesbacher S, Pfitzer L, Wiedorfer K, Angermeyer S, Borst A, Haferkamp S, et al. RB1 is the crucial target of the Merkel cell polyomavirus Large T antigen in Merkel cell carcinoma cells. *Oncotarget.* 2016;7(22):32956-68.
24. Anders S, Pyl PT, and Huber W. HTSeq--a Python framework to work with high-throughput sequencing data. *Bioinformatics (Oxford, England).* 2015;31(2):166-9.
25. Patro R, Duggal G, Love MI, Irizarry RA, and Kingsford C. Salmon provides fast and bias-aware quantification of transcript expression. *Nature methods.* 2017;14(4):417-9.
26. Love M, Soneson C, and Patro R. Swimming downstream: statistical analysis of differential transcript usage following Salmon quantification. *F1000Research.* 2018;7:952.
27. Yoshihara K, Shahmoradgoli M, Martínez E, Vegesna R, Kim H, Torres-Garcia W, et al. Inferring tumour purity and stromal and immune cell admixture from expression data. *Nat Commun.* 2013;4:2612.
28. Gartin AK, Frost TC, Cushman CH, Leeper BA, Gokhale PC, DeCaprio JA. Merkel cell carcinoma sensitivity to EZH2 inhibition is mediated by SIX1 derepression. *Journal of Investigative Dermatology.* In Press.
