# Supplementary material for: Reversal of viral and epigenetic HLA class I repression in Merkel cell carcinoma
Source: J Clin Invest. 2022 Jul 1;132(13):e151666. doi: 10.1172/JCI151666 (PMC9246387; doi:10.1172/JCI151666)
Supplement: Supplemental tables 6-7 [file jci-132-151666-s200.pdf]

Patient	HLA Allele	Tumor		Cell Line	
MCC-277	HLA-A	HLA-A*11:01:01	HLA-A*32:01:01	HLA-A*11:01:01	HLA-A*32:01:01
	HLA-B	HLA-B*14:01:01	HLA-B*51:01:01	HLA-B*14:01:01	HLA-B*51:01:01
	HLA-C	HLA-C*15:02:01	HLA-C*08:02:01	HLA-C*15:02:01	HLA-C*08:02:01
MCC-301	HLA-A	HLA-A*24:02:01:01	HLA-A*02:01:01:01	HLA-A*24:02:01:01	HLA-A*02:01:01:01
	HLA-B	HLA-B*15:18:01	HLA-B*44:02:01:01	HLA-B*15:18:01	HLA-B*44:02:01:01
	HLA-C	HLA-C*07:04:01	HLA-C*05:01:01:02	HLA-C*07:04:01	HLA-C*05:01:01:02
MCC-320	HLA-A	HLA-A*01:01:01:01	HLA-A*25:01:01	HLA-A*01:01:01:01	HLA-A*25:01:01
	HLA-B	HLA-B*14:01:01	HLA-B*18:01:01:02	HLA-B*14:01:01	HLA-B*18:01:01:02
	HLA-C	HLA-C*12:03:01:01	HLA-C*08:02:01	HLA-C*12:03:01:01	HLA-C*08:02:01
MCC-336	HLA-A	HLA-A*02:01:01:01	HLA-A*02:01:01:01	HLA-A*02:01:01:01	HLA-A*02:01:01:01
	HLA-B	HLA-B*35:02:01	HLA-B*52:01:01:02	HLA-B*35:02:01	HLA-B*52:01:01:02
	HLA-C	HLA-C*12:02:02	HLA-C*04:01:01:01	HLA-C*12:02:02	HLA-C*04:01:01:01
MCC-350	HLA-A	HLA-A*24:02:01:01	HLA-A*29:02:01:01	HLA-A*24:02:01:01	HLA-A*29:02:01:01
	HLA-B	HLA-B*07:02:01	HLA-B*08:01:01	HLA-B*07:02:01	HLA-B*08:01:01
	HLA-C	HLA-C*07:02:01:01	HLA-C*07:01:01:01	HLA-C*07:02:01:01	HLA-C*07:01:01:01

MCC-367	HLA-A	HLA-A*01:01:01:01	HLA-A*31:01:02	HLA-A*01:01:01:01	HLA-A*31:01:02
	HLA-B	HLA-B*49:01:01	HLA-B*51:01:01	HLA-B*49:01:01	HLA-B*51:01:01
	HLA-C	HLA-C*12:03:01:01	HLA-C*01:02:01	HLA-C*12:03:01:01	HLA-C*01:02:01
MCC-2314	HLA-A	HLA-A*24:02:01:01	HLA-A*02:01:01:01	HLA-A*24:02:01:01	HLA-A*02:01:01:01
	HLA-B	HLA-B*07:02:01	HLA-B*44:02:01:01	HLA-B*07:02:01	HLA-B*44:02:01:01
	HLA-C	HLA-C*07:02:01:03	HLA-C*05:01:01:02	HLA-C*07:02:01:03	HLA-C*05:01:01:02

Supplemental Table 6. HLA typing.
HLA typing for 7 of the 11 MCC lines for which whole-exome sequencing data was available.

Oligo Name	Sequence	Notes
BCORL1-1 fwd	CACCGTCCCGCATCTGACAGCGCCG	Oligo for guide RNA cloning
BCORL1-1 rev	AAACCGGCGCTGTCAGATGCGGGAC	Oligo for guide RNA cloning
BCORL1-2 fwd	CACCGGGAGGCGGGATATATACCAG	Oligo for guide RNA cloning
BCORL1-2 rev	AAACCTGGTATATATCCCGCCTCCC	Oligo for guide RNA cloning
USP7-1 fwd	CACCGTTGATGACGACGTGGTGTCA	Oligo for guide RNA cloning
USP7-1 rev	AAACTGACACCACGTCGTCATCAAC	Oligo for guide RNA cloning
USP7-2 fwd	CACCGGGCAGTAGAACAGCTCGATG	Oligo for guide RNA cloning
USP7-2 rev	AAACCATCGAGCTGTTCTACTGCCC	Oligo for guide RNA cloning
PCGF1-1 fwd	CACCGCCACGAAGTAGCCGGCGCAT	Oligo for guide RNA cloning
PCGF1-1 rev	AAACATGCGCCGGCTACTTCGTGGC	Oligo for guide RNA cloning
PCGF1-2 fwd	CACCGGCTCATCATAGCGATAGTAG	Oligo for guide RNA cloning
PCGF1-2 rev	AAACCTACTATCGCTATGATGAGCC	Oligo for guide RNA cloning
CTRL-1 fwd	CACCGTGCGGCGTAATGCTTGAAAG	Oligo for guide RNA cloning
CTRL-1 rev	AAACCTTTCAAGCATTACGCCGCAC	Oligo for guide RNA cloning
CTRL-2 fwd	CACCGGGATTAATTCGCTAAATGAT	Oligo for guide RNA cloning
CTRL-2 rev	AAACATCATTTAGCGAATTAATCCC	Oligo for guide RNA cloning
shMYCL fwd	CCGGACCAAGAGGAAGAATCACAATCAAGA GTTGTGATTCTTCCTCTTGGTCTTTTT	Oligo for shRNA
shMYCL rev	AATTAAAAAGACCAAGAGGAAGAATCACA ACTCTTGATTGTGATTCTTCCTCTTGG	Oligo for shRNA
shEP400-2 fwd	ccggCTGCGAAGAAGCTCGTTAGATCAAGAG TCTAACGAGCTTCTTCGCAGCttttt	Oligo for shRNA
shEP400-2 rev	aattAAAAAGCTGCGAAGAAGCTCGTTAGACT CTTGATCTAACGAGCTTCTTCGCAG	Oligo for shRNA
shEP400-3 fwd	ccggAGCAGCTTACACCAATTGAtcaagagTCAA TTGGTGTAAGCTGCTCCttttt	Oligo for shRNA
shEP400-3 rev	aattAAAAAGGAGCAGCTTACACCAATTGACT CTTGATCAATTGGTGTAAGCTGCT	Oligo for shRNA
LentiCRISPRv2 sequencing primer	GATACAAGGCTGTTAGAGAGATAATT	
USP7 ChIP fwd	CCAACGACCAACTCCCTAAAT	ChIP-qPCR primer
USP7 ChIP rev	AAGGCACTGTAGTTTGAGGTATAG	ChIP-qPCR primer
PCGF1 ChIP fwd	TCGCCTCCTTCATCACACTA	ChIP-qPCR primer
PCGF1 ChIP rev	CGAGTCCACGTGAGGGAA	ChIP-qPCR primer
Intergenic ChIP fwd	CTTCTTCCTTCCGGCTTTCT	ChIP-qPCR primer
Intergenic ChIP rev	AGCTGGGAGAGGACACACAC	ChIP-qPCR primer
USP7 qPCR primer fwd	GGCCGACACCAGTACATAAA	qPCR primer
USP7 qPCR primer rev	GGCCGAGGATGAGACATATTAC	qPCR primer
PCGF1 qPCR primer fwd	CCACTGCTCAACCTCAAACT	qPCR primer
PCGF1 qPCR primer rev	TTCCCGAATCCGTTTCTCTTC	qPCR primer
MYCL qPCR primer fwd	CGACTCGTACCAGCACTATTT	qPCR primer

MYCL qPCR primer rev	TGGCACCAGCTCGAATTT	qPCR primer
18S rRNA qPCR primer fwd	ACCCCGTTGAACCCCATT	qPCR primer
18S rRNA qPCR primer rev	CCATCCAATCGGTAGTAGCG	qPCR primer
NLRC5 qPCR primer A fwd	CCTGGATGCCGTGTTGGGTT	qPCR primer
NLRC5 qPCR primer A rev	GGCCCACATATCCCTGCTTGT	qPCR primer
NLRC5 qPCR primer B fwd	ATGTCGGTTGCTGCCACCTTT	qPCR primer
NLRC5 qPCR primer B rev	GGCATTGCAGCCAAGCATCAAC	qPCR primer
GAPDH primer fwd	TGCACCACCAACTGCTTAGC	qPCR primer
GAPDH primer rev	GGCATGGACTGTGGTCATGAG	qPCR primer

Supplemental Table 7. Oligos and primers.
